# Modeling precision treatment of breast cancer

**DOI:** 10.1186/gb-2013-14-10-r110

**Published:** 2013-10-31

**Authors:** Anneleen Daemen, Obi L Griffith, Laura M Heiser, Nicholas J Wang, Oana M Enache, Zachary Sanborn, Francois Pepin, Steffen Durinck, James E Korkola, Malachi Griffith, Joe S Hur, Nam Huh, Jongsuk Chung, Leslie Cope, Mary Jo Fackler, Christopher Umbricht, Saraswati Sukumar, Pankaj Seth, Vikas P Sukhatme, Lakshmi R Jakkula, Yiling Lu, Gordon B Mills, Raymond J Cho, Eric A Collisson, Laura J van’t Veer, Paul T Spellman, Joe W Gray

**Affiliations:** 1 Department of Cancer & DNA Damage Responses, Life Sciences Division, Lawrence Berkeley National Laboratory, Berkeley, CA 94720, USA; 2 Laboratory Medicine, University of California San Francisco, San Francisco, CA 94115, USA; 3 Department of Molecular and Medical Genetics, Oregon Health and Science University, Portland, OR 97239, USA; 4 Department of Biomedical Engineering, Center for Spatial Systems Biomedicine, Knight Cancer Institute, Oregon Health and Science University, Portland, OR 97239, USA; 5 Five3 genomics, 101 Cooper St, Santa Cruz, CA 95060, USA; 6 The Genome Institute, Washington University School of Medicine, St Louis, MO 63105, USA; 7 Samsung Electronics Headquarters, Seocho-gu, Seoul 137-857, Korea; 8 Emerging Technology Research Center, Samsung Advanced Institute of Technology, Kyunggi-do 446-712, Korea; 9 Department of Oncology, Johns Hopkins University School of Medicine, Baltimore, MD 21205, USA; 10 Department of Medicine, Beth Israel Deaconess Medical Center, Harvard Medical School, Boston, MA 02215, USA; 11 Department of Systems Biology, MD Anderson Cancer Center, Houston, TX 77030, USA; 12 Department of Dermatology, University of California, San Francisco, CA 94115, USA; 13 Present address: Department of Bioinformatics & Computational Biology, Genentech Inc, 1 DNA Way, South San Francisco, CA 94080, USA; 14 Present address: Sequenta Inc, South San Francisco, CA 94080, USA

## Abstract

**Background:**

First-generation molecular profiles for human breast cancers have enabled the identification of features that can predict therapeutic response; however, little is known about how the various data types can best be combined to yield optimal predictors. Collections of breast cancer cell lines mirror many aspects of breast cancer molecular pathobiology, and measurements of their omic and biological therapeutic responses are well-suited for development of strategies to identify the most predictive molecular feature sets.

**Results:**

We used least squares-support vector machines and random forest algorithms to identify molecular features associated with responses of a collection of 70 breast cancer cell lines to 90 experimental or approved therapeutic agents. The datasets analyzed included measurements of copy number aberrations, mutations, gene and isoform expression, promoter methylation and protein expression. Transcriptional subtype contributed strongly to response predictors for 25% of compounds, and adding other molecular data types improved prediction for 65%. No single molecular dataset consistently out-performed the others, suggesting that therapeutic response is mediated at multiple levels in the genome. Response predictors were developed and applied to TCGA data, and were found to be present in subsets of those patient samples.

**Conclusions:**

These results suggest that matching patients to treatments based on transcriptional subtype will improve response rates, and inclusion of additional features from other profiling data types may provide additional benefit. Further, we suggest a systems biology strategy for guiding clinical trials so that patient cohorts most likely to respond to new therapies may be more efficiently identified.

## Background

Breast cancer is a clinically and genomically heterogeneous disease. Six subtypes were defined approximately a decade ago based on transcriptional characteristics and were designated luminal A, luminal B, ERBB2-enriched, basal-like, claudin-low and normal-like [1,2]. New cancers can be assigned to these subtypes using a 50-gene transcriptional signature designated the PAM50 [1]. However, the number of distinct subtypes is increasing steadily as multiple data types are integrated. Integration of genome copy number and transcriptional profiles defines 10 subtypes [3], and adding mutation status [4], methylation pattern [5], pattern of splice variants [6], protein and phosphoprotein expression [7] and microRNA expression and pathway activity [8] may define still more subtypes. The Cancer Genome Atlas (TCGA) project and other international genomics efforts were founded to improve our understanding of the molecular landscapes of most major tumor types with the ultimate goal of increasing the precision with which individual cancers are managed. One application of these data is to identify molecular signatures that can be used to assign specific treatment to individual patients. However, strategies to develop optimal predictive marker sets are still being explored. Indeed, it is not yet clear which molecular data types (genome, transcriptome, proteome, and so on) will be most useful as response predictors.

In breast cancer, cell lines mirror many of the molecular characteristics of the tumors from which they were derived, and are therefore a useful preclinical model in which to explore strategies for predictive marker development [8,9]. To this end, we have analyzed the responses of 70 well characterized breast cancer cell lines to 90 compounds and used two independent machine learning approaches to identify pretreatment molecular features that are strongly associated with responses within the cell line panel. For most compounds tested, *in vitro* cell line systems provide the only experimental data that can be used to identify predictive response signatures, as most of the compounds have not been tested in clinical trials. Our study focuses on breast cancer [10,11] and extends earlier efforts [12-14], by including more cell lines, by evaluating a larger number of compounds relevant to breast cancer, and by increasing the molecular data types used for predictor development. Data types used for correlative analysis include pretreatment measurements of mRNA expression, genome copy number, protein expression, promoter methylation, gene mutation, and transcriptome sequence (RNAseq). This compendium of data is now available to the community as a resource for further studies of breast cancer and the inter-relationships between data types. We report here on initial machine learning-based methods to identify correlations between these molecular features and drug response. In the process, we assessed the utility of individual data sets and the integrated data set for response predictor development. We also describe a publicly available software package that we developed to predict compound efficacy in individual tumors based on their omic features. This tool could be used to assign an experimental compound to individual patients in marker-guided trials, and serves as a model for how to assign approved drugs to individual patients in the clinical setting. We explored the performance of the predictors by using it to assign compounds to 306 TCGA samples based on their molecular profiles.

## Results and discussion

### Breast cancer cell line panel

We assembled a collection of 84 breast cancer cell lines composed of 35 luminal, 27 basal, 10 claudin-low, 7 normal-like, 2 matched normal cell lines, and 3 of unknown subtype (Additional file 1) [8]. Fourteen luminal and 7 basal cell lines were also ERBB2-amplified. Seventy cell lines were tested for response to 138 compounds by growth inhibition assays. The cells were treated in triplicate with nine different concentrations of each compound as previously described [8]. The concentration required to inhibit growth by 50% (GI_50_) was used as the response measure for each compound. Compounds with low variation in response in the cell line panel were eliminated, leaving a response data set of 90 compounds. An overview of the 70 cell lines with subtype information and 90 therapeutic compounds with GI_50_ values is provided in Additional file 1. All 70 lines were used in development of at least some predictors depending on data type availability. The therapeutic compounds include conventional cytotoxic agents such as taxanes, platinols and anthracyclines, as well as targeted agents such as hormone and kinase inhibitors. Some of the agents target the same protein or share common molecular mechanisms of action. Responses to compounds with common mechanisms of action were highly correlated, as has been described previously [8].

### A rich and multi-omic molecular profiling dataset

Seven pretreatment molecular profiling data sets were analyzed to identify molecular features associated with response. These included profiles for DNA copy number (Affymetrix SNP6 - EGA accessions EGAS00000000059 and EGAS00001000585), mRNA expression (Affymetrix U133A and Exon 1.0 ST array - ArrayExpress accessions E-TABM-157 and E-MTAB-181), transcriptome sequence (RNAseq - Gene Expression Omnibus (GEO) accession GSE48216), promoter methylation (Illumina Methylation27 BeadChip - GEO accession GSE42944), protein abundance (Reverse Protein Lysate Array - Additional file 2), and mutation status (Exome-Seq - GEO accession GSE48216). The data were preprocessed as described in Supplementary Methods of Additional file 3. Figure S1 in Additional file 3 gives an overview of the number of features per data set before and after filtering based on variance and signal detection above background where applicable. Exome-seq data were available for 75 cell lines, followed by SNP6 data for 74 cell lines, therapeutic response data for 70, RNAseq for 56, exon array for 56, Reverse Phase Protein Array (RPPA) for 49, methylation for 47, and U133A expression array data for 46 cell lines. Information on the overlap in cell lines with both response data and molecular data is provided in Additional file 3. The set of 48 core cell lines was defined as those with response data and at least 4 molecular data sets.

### Inter-data relationships

We investigated the association between expression, copy number and methylation data. We distinguished correlation at the cell line level and gene level. At the cell line level, we report average correlation between datasets for each cell line across all genes, while correlation at the gene level represents the average correlation between datasets for each gene across all cell lines. Correlation among the three expression datasets (U133A, exon array, and RNAseq) ranged from 0.6 to 0.77 at the cell line level, and from 0.58 to 0.71 at the gene level. Promoter methylation and gene expression were, on average, negatively correlated as expected, with correlation ranging from -0.16 to -0.25 at the cell line level and -0.10 to -0.15 at the gene level. Across the genome, copy number and gene expression were positively correlated (0.18 to 0.22 at the cell line level; 0.35 to 0.44 at the gene level). When restricted to copy number aberrations, 22 to 39% of genes in the aberrant regions showed a significant concordance between their genomic and transcriptomic profiles from U133A, exon array and RNAseq after multiple testing correction (see the 'Intra-data relationships’ section in Supplementary Results in Additional file 3 and Table S4a-c in Additional file 3).

### Machine learning approaches identify accurate cell line-derived response signatures

**Figure 1:**
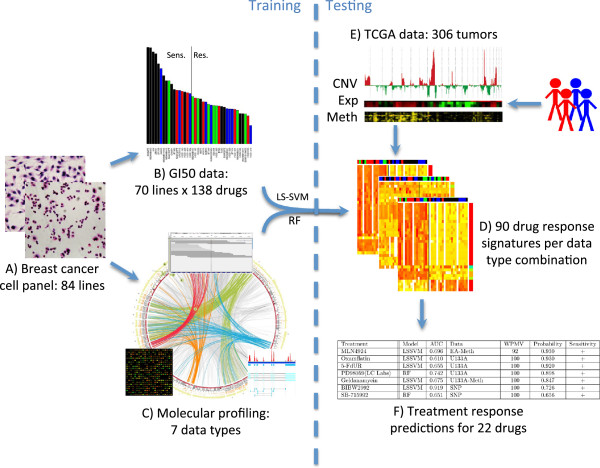
**Cell line-based response prediction strategy. (A)** We assembled a collection of 84 breast cancer cell lines composed of 35 luminal, 27 basal, 10 claudin-low, 7 normal-like, 2 matched normal and 3 of unknown subtype. Fourteen luminal and 7 basal cell lines were also ERBB2-amplified. **(B)** Seventy lines were tested for response to 138 compounds by growth inhibition assays. Compounds with low variation in response in the cell line panel were eliminated, leaving a response data set of 90 compounds. Cell lines were divided into a sensitive and resistant group for each compound using the mean GI_50_ value for that compound. **(C)** Seven pretreatment molecular profiling data sets were analyzed to identify molecular features associated with response. Exome-seq data were available for 75 cell lines, followed by SNP6 data for 74 cell lines, RNAseq for 56, exon array for 56, RPPA for 49, methylation for 47, and U133A expression array data for 46 cell lines. All 70 lines were used in development of at least some predictors depending on data type availability. **(D)** Classification signatures were developed using the molecular feature data (after filtering) and with response status as the target. Two methods, weighted least squares support vector machine (LS-SVM) and random forests (RF), were utilized. The best performing signature was chosen for each drug and data type combination. This allows prediction of response for additional cell lines or tumors with any given combination of input data types. **(E)** Cell line-based response predictors were applied to 306 TCGA breast tumors for which expression (Exp), copy number (CNV) and methylation (Meth) measurements were all available. **(F)** This identified 22 compounds with a model AUC >0.7 for which at least some patients were predicted to be responsive with a probability >0.65. Thresholds for considering a tumor responsive were objectively chosen for each compound from the distribution of predicted probabilities and each patient was assigned to a status of resistant, intermediate or sensitive. WPMV, weighted percent of model variables.

We developed candidate response signatures by analyzing associations between biological responses to therapy and pretreatment omic signatures. We used the integrative approach displayed in Figure 1 for the construction of compound sensitivity signatures. Standard data pre-processing methods were applied to each dataset. Classification signatures for response were developed using the weighted least squares support vector machine (LS-SVM) [15] in combination with a grid search approach for feature optimization, as well as random forests (RF) [16], both described in detail in the Supplementary Methods in Additional file 3. For this, the cell lines were divided into a sensitive and resistant group for each compound using the mean GI_50_ value for that compound (Additional file 4). This seemed most reasonable after manual inspection, with concordant results obtained using TGI (concentration required to achieve total growth inhibition) as response measure. Multiple random divisions of the cell lines into two-thirds training and one-third test sets were performed for both methods, and area under a receiver operating characteristic curve (AUC) was calculated as an estimate of accuracy (Additional file 3).

The candidate signatures incorporated copy number, methylation, transcription and/or proteomic features. We also included the mutation status of *TP53*, *PIK3CA*, *MLL3*, *CDH1*, *MAP2K4*, *PTEN* and *NCOR1*, chosen based on reported frequencies from TCGA breast project. That project sequenced the exomes of 507 breast invasive carcinomas and identified approximately 30,000 somatic mutations [4]. Each of the 7 genes was mutated in at least 3% of samples with a false discovery rate (FDR) *P*-value <0.05. Our whole exome sequencing showed that these genes were also mutated in at least 3% of the breast cancer cell lines. Their mutation rate in TCGA and the cell line panel showed a similar distribution across the subtypes (Figure S2 in Additional file 3). We excluded lower prevalence mutations because their low frequency limits the possibility of significant associations.

These signatures incorporating any of the molecular features are shown in Additional file 5. They predicted compound response within the cell lines with high estimated accuracy (AUC >0.70) regardless of classification method for 51 (57%) of the compounds tested. Concordance between GI_50_ and TGI exceeded 80% for 67% (34/51) of these compounds. A comparison across all 90 compounds of the LS-SVM and RF models with highest AUC based on copy number, methylation, transcription and/or proteomic features revealed a high correlation between both classification methods (Spearman correlation coefficient = 0.85, *P*-value <0.001), with the LS-SVM more predictive for 35 compounds and RF for 55 compounds (Figure S3 in Additional file 3). However, there was a better correlation between both classification methods for compounds with strong biomarkers of response (upper third; Spearman correlation coefficient 0.84) and compounds without a clear signal associated with drug response (lower third; Spearman correlation coefficient 0.46). This suggests that for compounds with strong biomarkers, a signature can be identified by either approach. For compounds with a weaker signal of drug response (middle third), there was a larger discrepancy in performance between both classification methods (Spearman correlation coefficient 0.16), with neither of them outperforming the other.

**Figure 2:**
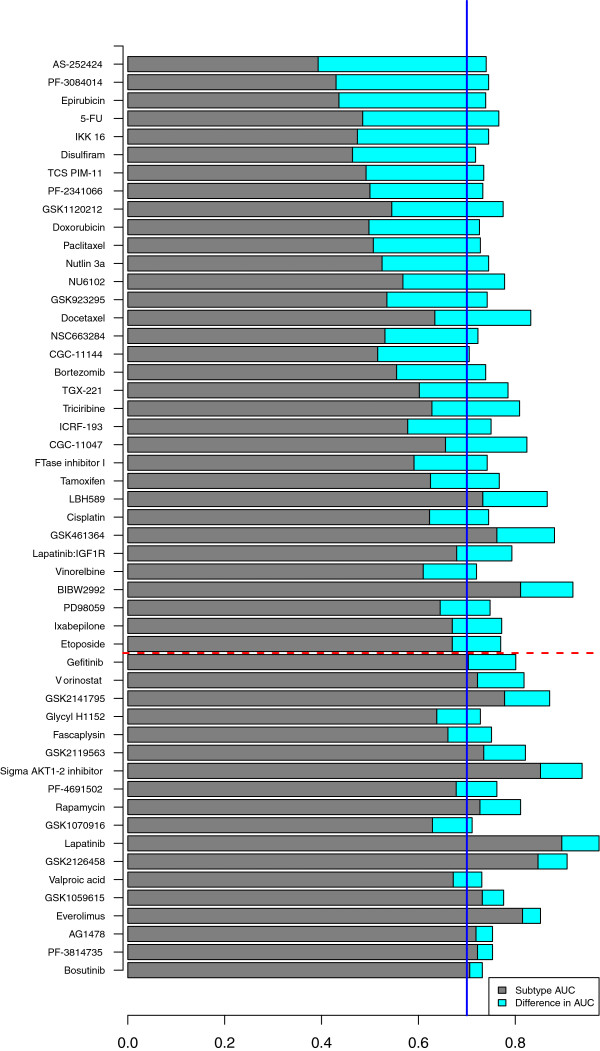
**Comparison of transcriptional subtype and molecular profiling for 51 (57%) of the compounds with predicted compound response within the cell lines with high estimated accuracy (AUC >0.70).** AUC obtained with transcriptional subtype is shown in gray. Compounds are ordered based on increase in AUC from subtype to the best performing molecular data. The increase in AUC with respect to subtype obtained with the best performing molecular data is shown in cyan. For 65% of the compounds, molecular profiling performed substantially better than subtype, with an AUC increase of at least 0.1 (compounds above the red dashed line). Subtype was sufficient for 25% of the compounds with AUC >0.70 and AUC increase obtained with molecular profiling less than 0.1 (compounds below the red dashed line with subtype AUC above the blue solid line).

Thirteen of the 51 compounds (25%) showed a strong transcriptional subtype-specific response (AUC >0.70), with the best omics signature not adding predictive information beyond a simple transcriptional subtype-based prediction (AUC increase below 0.1) (Figure 2; Additional file 5). This suggests that the use of transcriptional subtype alone could greatly improve prediction of response for a substantial fraction of agents [8], as is already done for the estrogen receptor (ER), ERBB2 receptor, and selective use of chemotherapy in breast cancer subtypes. This is consistent with our earlier report that molecular pathway activity varies between transcriptional subtypes [8]. However, deeper molecular profiling added significant predictive information about probable response for the majority of compounds (33/51 = 65%) with an increase in AUC of at least 0.1 beyond subtype alone. Mutation status of the seven genes introduced above was in general not more predictive than any other dataset, with the exception of tamoxifen and CGC-11144. For tamoxifen response, prediction based on mutation status was substantially better than subtype, driven predominantly by the higher mutation prevalence of *PIK3CA* mutations in luminal compared to basal breast cancer and therefore an association of *PIK3CA* mutation with lack of response [4]. For CGC-11144, the mutation-based AUC was 0.70, primarily driven by *TP53* and much higher than obtained with the best performing molecular data set (methylation, AUC 0.42).

### *In vivo* validation of the cell line-derived response signatures

**Figure 3:**
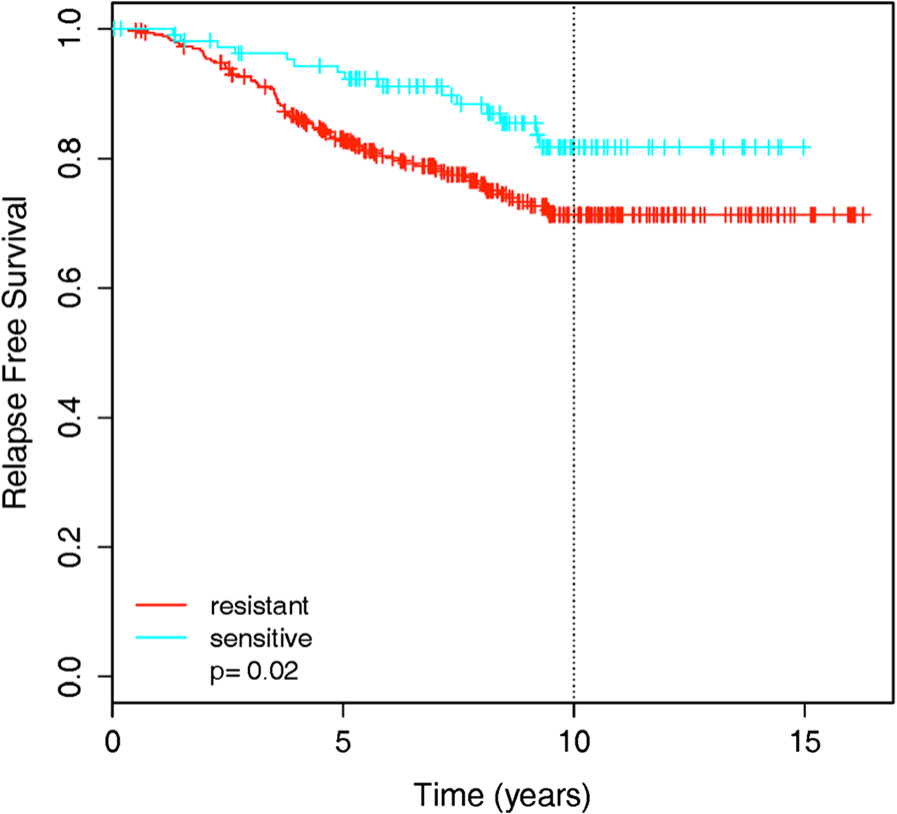
**Validation of the cell line signature for tamoxifen in a meta-set of 439 breast cancer patients treated with tamoxifen.** Kaplan-Meier plot of relapse free survival for patients predicted to be sensitive versus resistant to tamoxifen according to the 174-gene cell line-based predictor.

We validated *in vitro* signatures for expression profiles from tumor samples with response information, in addition to an assessment of cell line signal in tumor samples (Supplementary Results in Additional file 3). Such independent information was available for tamoxifen [17-20] and the histone deacetylase inhibitor valproic acid [21]. The independent tamoxifen data are from a meta-analysis where relapse-free survival status was available for 439 ER-positive patients [17-20]. Our *in vitro* 174-gene signature for tamoxifen, built on the complete panel of cell lines regardless of ER status, predicted a significantly improved relapse-free survival for patients predicted to be tamoxifen-sensitive (log-rank test; *P*-value 0.02; Figure 3). For valproic acid, therapeutic responses were tested for 13 tumor samples grown in three-dimensional cultures [21]. Our *in vitro* 150-gene signature for the histone deacetylase inhibitor vorinostat (Figure S4a in Additional file 3) distinguished valproic acid responders from non-responders (AUC = 0.97), with 7/8 sensitive samples (87.5%) and 4/5 resistant samples (80%) classified correctly when using a probability threshold of 0.5 for response dichotomization (Figure S4b in Additional file 3).

Unfortunately, omic profiles and corresponding clinical responses are not available for the other compounds tested *in vitro*. For these, we investigated whether the *in vitro* predictive signature was present in 536 breast TCGA tumors and consistent with the signature observed in cell lines. Here, we limited our analyses to those data types that are available in the TCGA dataset. Specifically, we developed response predictors for the breast cancer cell line panel using profiles for expression (U133A, exon array at the gene level, or RNAseq at the gene level), copy number, and promoter methylation for 51 compounds for which predictive power was high (AUC >0.7; Additional file 5). We applied these signatures to a set of 369 luminal, 95 basal, 8 claudin-low, and 58 ERBB2-amplified samples from the TCGA project. We used profiles of expression (n = 536), copy number (n = 306) and promoter methylation (n = 318) in our analyses. Additional file 5 shows that the transcriptional subtype specificities measured for these compounds in the cell lines were concordant with the subtype of TCGA samples predicted to respond. Figure S5 in Additional file 3 shows the predicted probability of response to four compounds with test AUC >0.7 for TCGA tumor samples ordered according to increasing probability. Importantly, genes in these signatures that were coordinately regulated in the set of cell lines were also coordinately regulated in the tumor samples (average Jaccard coefficient = 0.68, *P*-value <0.0001; Figure S6 in Additional file 3). This panel of 51 compounds represented most major therapeutic target classes (phosphatidylinositol 3-kinase (PI3K), receptor tyrosine kinase, anti-mitotic, DNA damage, cell cycle, proteasome, anti-metabolite, TP53, mitogen-activated protein kinase (MAPK), and estrogen antagonist). Eighteen of these compounds have been approved by the US Food and Drug Administration, including five for breast cancer. Phase I clinical trials are ongoing for seven compounds, phase II trials are underway for seven compounds, including six for breast cancer, and one compound is currently being tested in a phase III trial (Additional file 5). Thus further validation of signatures may be possible in the near future.

### Robust predictors of drug response are found at all levels of the genome

**Figure 4:**
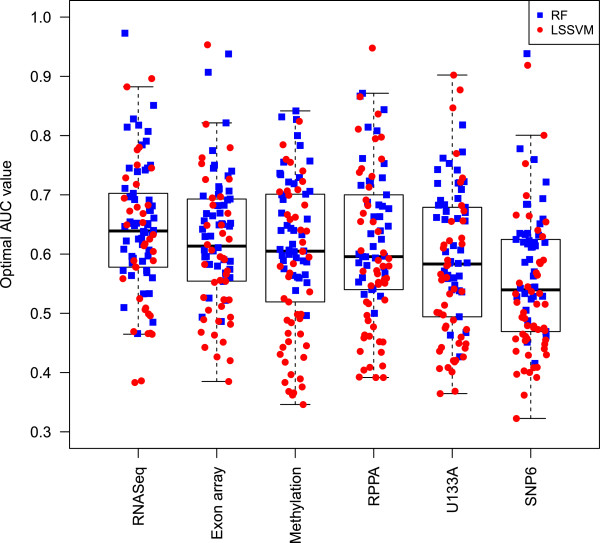
**Boxplot of best AUC values for all 90 compounds across 6 data types.** For all data types, the highest AUC obtained with either approach (LS-SVM in red circles, or random forest in blue squares) is displayed. For RNAseq and exon array, the highest AUC is shown among models built on gene-level data only versus all features (exons, junctions, and so on). The one-way repeated measures ANOVA test revealed a significant difference in performance among any of the data types (*P*-value 2.6e-5). *Post hoc* pairwise comparisons with multiple testing correction revealed a significant outperformance of RNAseq with respect to all other data types. SNP6 copy number performed significantly worse compared to all other data types, and exon array additionally significantly outperformed U133A.

With seven data types available on a single set of samples, we were well-positioned to assess whether particular technologies or molecular data types consistently out-perform others in the prediction of drug sensitivity. To obtain a ranking of the importance of the molecular datasets, we compared prediction performance of classifiers built on individual data sets and their combination for 29 common cell lines (with the exclusion of the normal-like cell lines). Importantly, no single data type performed well for all compounds, with each data type performing best for some compounds (Figure S7 in Additional file 3). Table S6a,c in Additional file 3 shows the ranking of the datasets according to the independent classifiers obtained with LS-SVM and RF, respectively. For the LS-SVM classifiers, RNAseq performed best for 22 compounds, exon array for 20 compounds, SNP6 for 18, U133A for 17 and methylation data for 12 compounds (Table S6a in Additional file 3). Similar results were confirmed with the RF approach (Table S6c in Additional file 3). Even though it had varying performance for individual compounds, in general, RNAseq significantly outperformed all other data types across the complete panel of 90 compounds (paired *t*-test with multiple testing correction; *P*-values ranging from 0.026 to 2e-8; Figure 4). SNP6 copy number data resulted in significantly worse predictive power compared to all other data types (*P*-values 0.038 to 2e-8). In addition, exon array outperformed U133A, with a *P*-value of 0.0002.

In Table S6b,d in Additional file 3, a distinction is made between two groups of compounds: compounds for which all datasets perform similarly well (for example, CGC-11047, GSK461364, GSK2126458, lapatinib) versus compounds for which results with one dataset are much better than obtained with any of the other datasets, defined as an AUC increase of at least 0.1. For example, exon array worked best for VX-680 (AUC 0.81), RNAseq for carboplatin (AUC 0.89), and RPPA for bortezomib (AUC 0.87). Data type specificity was in general not related to therapeutic compound class, although there were a few exceptions for LS-SVM with RNAseq performing well for polyamine analogs (CGC-11047, CGC-11144) and mitotic inhibitors (ixabepilone, paclitaxel, vinorelbine), SNP6 for ERBB2/epidermal growth factor receptor (EGFR) inhibitors (AG1478, BIBW2992, erlotinib, gefitinib, lapatinib), and methylation for CDK1 inhibitors (NU6102, purvalanol A). The full combination of genome-wide datasets yielded a higher AUC value than the best performing individual dataset for only a limited number of compounds (AKT1-2 inhibitor, GSK461364 and PF-4691502). The full combination signatures, however, generally ranked closely to the best signatures based on individual data types. We refer to the 'Robust predictors of drug response' section in Supplementary Results in Additional file 3 for two additional complementary analyses on dataset comparison.

### Splice-specific predictors provide only minimal information

We compared the performance of classifiers between the fully featured data and gene-level data in order to investigate the contribution of splice-specific predictors for RNAseq and exon array data. The fully featured data included transcript- and exon-level estimates for the exon array data and transcript-, exon-, junction, boundary-, and intron-level estimates for the RNAseq data. Overall, there was no increase in performance for classifiers built with 'splice-aware' data versus gene level only. The overall difference in AUC from all features minus gene-level was 0.002 for RNAseq and -0.006 for exon array, a negligible difference in both cases. However, there were a few individual compounds with a modest increase in performance when considering splicing information (Table S8 in Additional file 3). Interestingly, both ERBB2 targeting compounds, BIBW2992 and lapatinib, showed improved performance using splice-aware features in both RNAseq and exon array datasets. This suggests that splice-aware predictors may perform better for prediction of ERBB2 amplification and response to compounds that target it. However, the overall result suggests that prediction of response does not benefit greatly from splicing information over gene-level estimates of expression. This indicates that the high performance of RNAseq for discrimination may have more to do with that technology’s improved sensitivity and dynamic range, rather than its ability to detect splicing patterns.

### Pathway overrepresentation analysis aids in interpretation of the response signatures

We surveyed the pathways and biological processes represented by genes for the 49 best-performing therapeutic response signatures incorporating copy number, methylation, transcription, and/or proteomic features (i.e., no mutation status) with AUC >0.7 (Additional file 5). For these compounds we created functionally organized networks with the ClueGO plugin in Cytoscape [22] using Gene Ontology (GO) categories and Kyoto Encyclopedia of Genes and Genomes (KEGG)/BioCarta pathways (Supplementary Methods in Additional file 3). Our previous work identified transcriptional networks associated with response to many of these compounds [8]. In this study, 5 to 100% (median 79%) of GO categories and pathways present in the predictive signatures were found to be significantly associated with drug response (FDR *P*-value <0.05). The majority of these significant pathways, however, were also associated with transcriptional subtype (17 to 100%, median 70%). These were filtered out to capture subtype-independent biology underlying each compound’s mechanism of action. The resulting non-subtype-specific pathways with FDR *P*-value <0.05 are shown in Additional file 6. Eighty-eight percent of the compounds for which we conducted pathway analysis were significantly associated with one or more GO category and 80% were significantly associated with one or more KEGG pathway. The most commonly identified KEGG pathways (six or more compounds) were hedgehog signaling, basal cell carcinoma, glycosphingolipid biosynthesis, ribosome, spliceosome and Wnt signaling. The most commonly identified GO processes (six or more compounds) also included many critical cancer pathways and processes, such as regulation of cell cycle, cell death, protein kinase activity, metabolism, TGFβ receptor signaling, cell-cell adhesion, microtubule polymerization, and Wnt receptor signaling. Many of these processes can be linked directly to the known mechanisms of action of their associated compounds. For example, the signature for docetaxel was significantly enriched for microtubule polymerization genes. Docetaxel is known to function by microtubule disassembly inhibition. Similarly, signatures for the AKT1/2 kinase inhibitor, bosutinib SRC kinase inhibitor, TCS PIM-11 kinase inhibitor and four PI3K inhibitors (GSK2119563, GSK2126458, PF-4691502, TGX-221) were all enriched in genes involved in the negative regulation of protein kinase activity. These kinase regulation genes tended to be consistently up-regulated or both methylated and down-regulated, depending on the therapeutic response signature. Many of the genes in this enriched gene set have well-described roles in modulation of the PI3K/MAPK cascades, including *ERRFI1*[23], *DUSP6/7/8*[24] and *SPRY1/2/4*[25]. In particular, we found that high expression of *GADD45A* was associated with resistance to GSK2126458, PF-4691502 and the AKT1/2 inhibitor, which is consistent with the observation that AKT inhibition modulates cell growth via activation of *GADD45A*[26]. The pan-PI3K targeting agent GSK2126458 is reported to function as a competitive ATP binding inhibitor and the signature for this compound was over-represented in ATP metabolic processes [27].

Genomic aberrations and transcriptomic/proteomic features played prominent roles in some of the candidate response signatures. For copy number aberrations, ERBB2 amplification was strongly associated with response to the ERBB2 targeting compounds lapatinib (two-sample *t*-test, *P*-value 2.1e-11) and BIBW2992 (1.6e-5) and to EGFR inhibitors AG1478 (2.5e-4) and gefitinib (9.5e-4). In addition to the association of overall mutation status with tamoxifen and CGC-11144 response discussed above, we also found several individual mutations to be relevant for treatment response. The presence of mutations in *TP53* was strongly associated with response to the PI3K inhibitor BEZ235, with 13/25 (52%) of the sensitive cell lines harboring *TP53* mutations compared to 3/19 (16%) for the resistant cell lines (Fisher’s exact test, *P*-value 0.025). This may be an indication of synthetic lethality resulting from BEZ235 inhibition of ATR (Ataxia telangiectasia and Rad3-related protein) leading to replicative stress in TP53-deficient cells [28]. Kim *et al*. [29] showed a similar trend in a study of 310 cell lines across multiple lineages in which co-mutation of *TP53* and *PIK3CA* was positively associated with response to BEZ235. In our study, mutation status for *PIK3CA* was associated with response to the PI3K inhibitor GSK1059615B, with 11/27 (41%) sensitive cell lines carrying *PIK3CA* mutations compared to 2/21 (10%) for resistant cell lines (*P*-value 0.022). These findings are consistent with recent clinical observations in patients with breast and gynecologic malignancies where treatment with similar agents resulted in response for 30% of patients with *PIK3CA* mutations compared to a response rate of 10% in wild-type *PIK3CA* patients [30].

### Response signature Toolbox to predict response in individual tumors

Our long-term goal is to develop a way to select therapeutic compounds most likely to be effective in an individual patient. A shorter-term goal is to test experimental compounds in patients that are most likely to be responsive. Both of these goals require a strategy to order compounds according to their predicted relative efficacy for individual patients. To this end, we developed software to rank order compounds for predicted efficacy in individual patients (see the 'Patient response prediction toolbox in R' section in Supplementary Results in Additional file 3). The software applies signatures of response developed *in vitro* to measurements of expression, copy number, and/or methylation for individual samples and produces a list of recommended treatments ranked according to predicted probability of response and *in vitro* GI_50_ dynamic range. For cases where several compounds are predicted to be equally effective, highest priority is assigned to the compound with highest GI_50_ dynamic range in the cell line panel.

**Figure 5:**
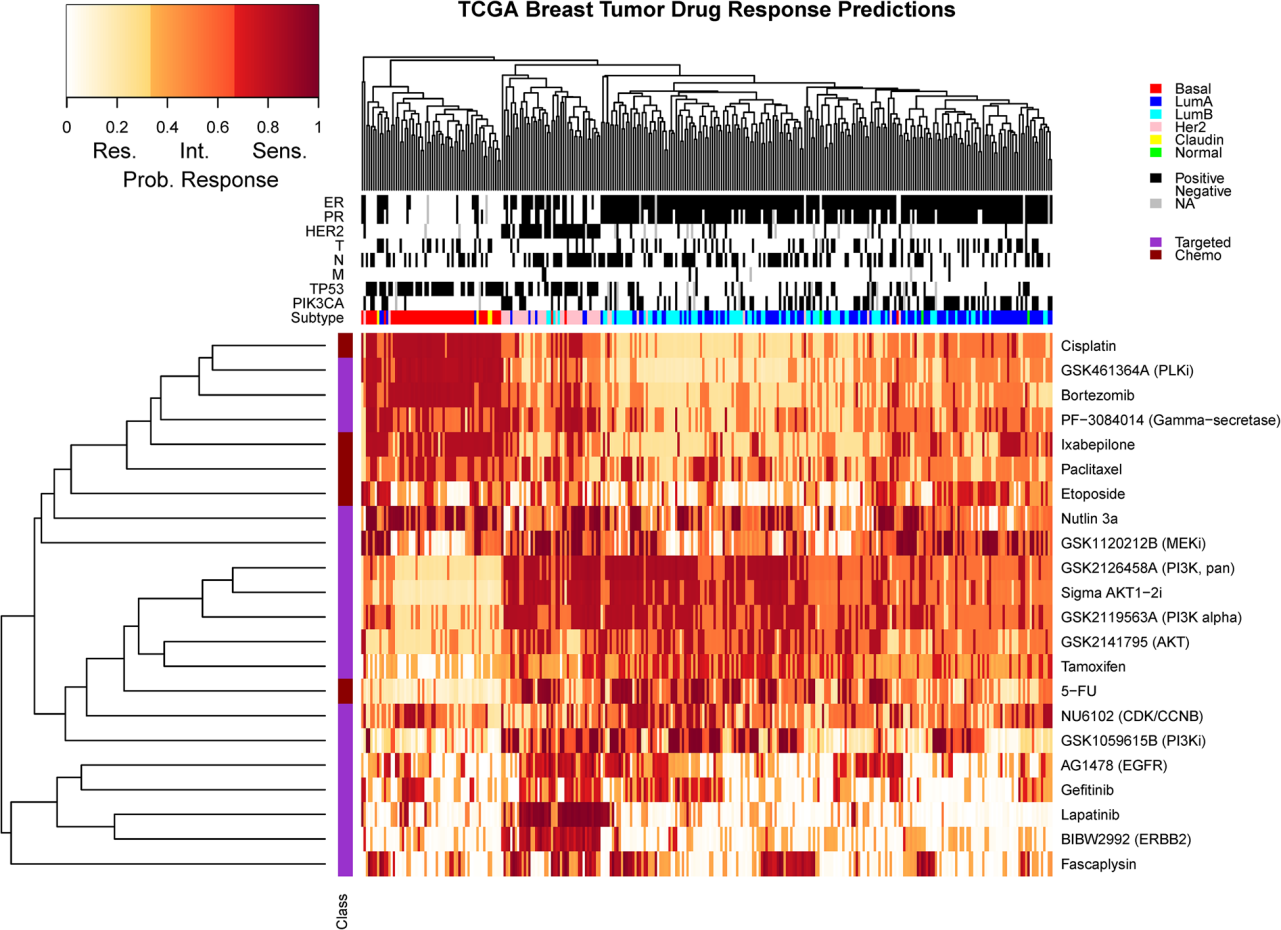
**Heatmap representation of predicted sensitivity in the TCGA population.** This heatmap represents the predicted pattern of sensitivity in 306 TCGA patients with expression, methylation and copy number data for 22 compounds with model AUC >0.7 and with at least some patients predicted to respond with probability >0.65. For the 306 patients, association is shown with ER, progesterone receptor (PR), ERBB2 status, tumor size (T), lymph node involvement (N), distant metastasis (M), and subtype. Of the 22 therapeutic compounds, 5 are chemotherapeutic and 17 are targeted agents. Probability values shown were re-scaled to reflect custom sensitivity thresholds as described in Supplementary Methods of Additional file 3. Res., resistant; Int., intermediate; Sens., sensitive.

Given the concordance of the predictive signatures for the 51 compounds in gene expression and subtype association between the cell lines and tumor samples from TCGA, we applied our *in vitro* response predictors to the 306 sample subset for which expression, copy number and methylation measurements were all available. This identified 22 compounds with a model AUC >0.7 for which at least some patients were predicted to be responsive with a probability >0.65. In all cases, thresholds for considering a tumor responsive were objectively chosen for each compound from the distribution of predicted probabilities and each patient was assigned to a status of resistant, intermediate or sensitive (see the 'Patient response prediction toolbox in R' section in Supplementary Results in Additional file 3, and Table S10 in Additional file 3). The resulting pattern of predicted sensitivity for the 22 compounds is displayed in Figure 5. Most of the compounds were predicted to have strong transcriptional subtype specificity (*P*-values 1.5e-70 to 0.02) although gefitinib and NU6102 were exceptions (Table S9 in Additional file 3). Not surprisingly, predicted sensitivity to lapatinib, BIBW2992 and to a lesser extent EGFR inhibitors was highly specific to ERBB2+ patients. Similarly, ER+ (and also many ERBB2+) patients were more frequently predicted to be sensitive to the PI3K inhibitors, AKT inhibitors, tamoxifen and to a lesser extent fluorouracil (5-FU). Patients in the basal (ER-/ERBB2-) subtype were predicted to be sensitive to cisplatin, PLK inhibitor (GSK461364A), bortezomib, gamma-secretase inhibitor (PF-3084014), paclitaxel and Nutlin 3A. The percentage of patients predicted to respond to any given compound ranged from 15.7% for BIBW2992 to 43.8% for the PI3K alpha inhibitor GSK2119563. Nearly all patients (99.3%) were predicted to respond to at least one treatment and each patient was predicted to be sensitive to an average of approximately six treatments. The predicted response rate to 5-FU was estimated at 23.9% (Figure S8 in Additional file 3), in agreement with the observed response rates to 5-FU as monotherapy in breast cancer (17% [21] to 26% [31,32]). The compound response signatures for the 22 compounds featured in Figure 5 are presented in Additional file 7.

## Conclusions

In this study we developed strategies to identify molecular response signatures for 90 compounds based on measured responses in a panel of 70 breast cancer cell lines, and we assessed the predictive strengths of several strategies. The molecular features comprising the high quality signatures are candidate molecular markers of response that we suggest for clinical evaluation. In most cases, the signatures with high predictive power in the cell line panel show significant PAM50 subtype specificity, suggesting that assigning compounds in clinical trials according to transcriptional subtype will increase the frequency of responding patients. However, our findings suggest that treatment decisions could further be improved for most compounds using specifically developed response signatures based on profiling at multiple omic levels, independent of - or in addition to - the previously defined transcriptional subtypes. We make available the drug response data and molecular profiling data from seven different platforms for the entire cell line panel as a resource for the community to aid in improving methods of drug response prediction.

We found predictive signatures of response across all platforms and levels of the genome. When restricting the analysis to just 55 well-known cancer proteins and phosphoprotein genes, all platforms do a reasonable job of measuring a signal associated with and predictive of drug response. This indicates that if a compound has a molecular signature that correlates with response, it is likely that many of the molecular data types will be able to measure this signature in some way. Furthermore, there was no substantial advantage of the combined platforms compared with the individual platforms. Some platforms might be able to measure the signature with slightly better accuracy, but our results indicate that many of the platforms could be optimized to identify a response-associated predictor.

Conversely, in the genome-wide comparison, the more comprehensive platforms are the ones that overall resulted in better prediction performance. This difference may reflect the fact that for those platforms, we selected the most significant feature per gene. For example, when a gene measured on the Affymetrix microarray is significantly differentially expressed, the chance is high that a particular exon or transcript is even more significant. Thus, the richness of data types like RNAseq offer the chance to identify both the signature and the most useful specific gene regions and junctions for use in a diagnostic (Figure 4). Taken together, these results suggest that the more comprehensive genome-wide platforms could be used for discovery, and once identified, significant features can be migrated to alternative platforms for a lab diagnostic.

Currently, treatment decisions are guided by ER and ERBB2 status. Using the TCGA dataset of 306 samples with expression, copy number and methylation measurements as a hypothetical example (Figure 5), a personalized treatment decision would be available for 81% of patients based on ERBB2 or ER status alone (55 ERBB2+, 193 ERBB2-/ER+). However, given reported response rates for trastuzumab (15 to 50%) [33] and tamoxifen (approximately 25%) [34] we can expect a substantial fraction of these will not respond. The candidate predictors proposed here could inform such clinical decisions for nearly all patients. Therefore, by considering diverse molecular data, we might suggest treatment options for not only the approximately 20% of patients who are ERBB2-/ER- but also secondary treatment options for those who will suboptimally respond to ER or ERBB2 directed treatments.

While our efforts to develop predictive drug response signatures are quite promising, they come with several conceptual caveats. Although the cell line panel is a reasonable model system, it does not capture several features known to be of critical importance in primary tumors. In particular, we have not modeled influences of the microenvironment, including additional cell types known to contribute to tumorigenesis [35], as well as variation in oxygen content, which has been shown to influence therapeutic response [36]. Expanding these experiments to three-dimensional model systems or mouse xenografts would aid in translation to the clinic. Additionally, validating these predictors in independent data sets will be important for determining how robust they are (see Supplementary Results and Additional file 8). Despite these limitations, our observation that we could find evidence of these predictive signatures in the TCGA data suggests that our cell line system is likely capturing many of the key elements involved in mediating therapeutic response.

Of course, the cell line-derived predictive signatures described in this study require substantial clinical validation. One possibility is in neoadjuvant trials like the I-SPY 2 TRIAL [37], in which *in vitro*-derived signatures for individual compounds are tested for power in predicting pathologic complete response or change in tumor volume measured with magnetic resonance imaging. An alternative approach for validation of signatures for approved drugs is to compare outcomes in patients assigned compounds according to *in vitro* predictors with outcomes in patients assigned drugs according to physicians’ first treatment choice. This study constitutes the basis for such a trial, with the development of a portfolio of *in vitro* predictors (for example, the 22 compounds displayed in Figure 5) and a computational tool that physicians might use to select compounds from that portfolio for individual patients.

Regardless of the specific design of the clinical trial, gene expression, methylation and copy number levels should be collected for all patients. High throughput sequencing techniques can provide all three with the additional benefits of alternative splicing information. As outlined in Figure 1, measurements of expression, methylation and copy number would serve as input to the predictor toolbox. The output of the toolbox consists of a report for each individualized patient, with the 22 therapeutic compounds ranked according to a patient’s likelihood of response and *in vitro* GI_50_ dynamic range. The full panel of 22 drug compounds could be tested simultaneously in a multi-arm trial to speed up the validation of the *in vitro* approach. The proposed clinical trial may also involve further optimizing of the number of markers in the signatures and choosing clinically relevant thresholds for tumor classification.

## Materials and methods

We refer to Supplementary Methods in Additional file 3 for a detailed description of the therapeutic compound response data, molecular data for the breast cancer cell lines, molecular data for the external breast cancer tumor samples used for validation, classification methods, data integration approach, statistical methods, pathway overrepresentation analysis, and the patient response prediction toolbox for the R project for statistical computing.

### Data and code deposition

Genome copy number data have been deposited at the European Genome-phenome Archive (EGA) [38], hosted at the EBI (accession numbers EGAS00000000059 and EGAS00001000585). Gene expression data for the cell lines were derived from Affymetrix GeneChip Human Genome U133A and Affymetrix GeneChip Human Exon 1.0 ST arrays. Raw data are available in ArrayExpress [39], hosted at the EBI (accession numbers E-TABM-157 and E-MTAB-181). RNAseq and exome-seq data can be accessed at the GEO, [40], accession number GSE48216. Genome-wide methylation data for the cell lines are also available through GEO, accession number GSE42944. Software and data for treatment response prediction are available on Synapse [41]. The software has also been deposited at GitHub [42]. The raw drug response data are available as Additional file 9.

## Abbreviations

5-FU: fluorouracil; AUC: area under the receiver operating characteristic curve; EGFR: epidermal growth factor receptor; ER: estrogen receptor; FDR: false discovery rate; GEO: Gene Expression Omnibus; GI50: concentration at which growth is inhibited by 50%; GO: Gene Ontology; KEGG: Kyoto Encyclopedia of Genes and Genomes; LS-SVM: least squares support vector machine; MAPK: mitogen-activated protein kinase; PAM: Prediction Analysis for Microarrays; PI3K: phosphatidylinositol 3-kinase; RF: random forests; RPPA: Reverse Phase Protein Array; SNP: single nucleotide polymorphism; TCGA: The Cancer Genome Atlas; TGI: total growth inhibition.

## Competing interests

The authors declare that they have no competing interests.

## Authors’ information

AD was partly supported by a BAEF Fellowship of the Belgian American Educational Foundation for postdoctoral research, OLG was supported by a Fellowship from the Canadian Institutes of Health Research, EAC is supported by NCI 5K08CA137153-02.

## Supplementary Material

Additional file 1 **Supplementary Methods, Supplementary Results, Figures S1 to S10, and Tables S4, S6, S8, S9, S10, S12, and S13. Supplementary Methods:** detailed description of the therapeutic compound response data, molecular data for the breast cancer cell lines, molecular data for the external breast cancer tumor samples used for validation, classification methods, data integration approach, statistical methods, and pathway overrepresentation analysis. **Supplementary Results:** assessment of cell line signal in tumor samples, inter-data relationships, prediction comparison of datasets, validation against other cell line datasets, and the patient response prediction toolbox for the R project for statistical computing. **Table S4:** overview of genes with good correlation (FDR *P*-value <0.05) between SNP6 and gene expression; 22 to 39% of genes in copy number aberration regions show a significant concordance between their genomic and transcriptomic profile after multiple testing correction. **Table S6:** data type ranking of the importance of the molecular datasets by comparison of prediction performance of LS-SVM and RF classifiers built on individual data sets and their combination, and by comparison of the average appearance of data types in the top 100 of ranked features, with and without inclusion of RPPA data. Examples are also provided of compounds for which (most) datasets give similar results or for which one dataset performs better (shown in bold). **Table S8:** performance for 'splice-specific' response predictors (RF) with an AUC increase >0.05 when comparing all transcript features to gene-level values alone. **Table S9:** statistical association between clinical variables and predicted response for 306 TCGA patients with expression, methylation and copy number data available. For each compound, the best performing model was utilized (LS-SVM or RF with any combination of expression, copy number and methylation data). **Table S10:** resistant/intermediate/sensitive cutoffs for 22 compounds with model AUC >0.7 and at least one patient with probability of response >0.65. Cutoff value 1 separates patients considered resistant from intermediate. Cutoff value 2 separates patients considered intermediate from sensitive. The percentage value for each group indicates the percentage of total patients (n = 306) in each group. **Table S12:** presence and variance of filtered features from U133A and exon array cell line data in tumor samples. Features from U133A and the exon array that passed the variance and presence filter in the cell lines were present in the majority of breast cancer tumor samples. **Table S13:** summary of 167 predictors in random forests classifier for lapatinib (all data types, optimal predictor number). **Figure S1:** data summary in terms of number of features before and after data-type-specific reduction and unsupervised filtering based on variance and signal detection above background. **Figure S2:** overview of the mutation prevalence in the cell line panel and TCGA data set for the list of seven common coding variants detected by TCGA, with a distinction between luminal, basal and ERBB2-enriched. Cell lines with unknown subtype are displayed in orange. To make the subtypes comparable, luminal A and B were grouped into luminal for the TCGA data set, whilst basal and claudin-low cell lines were grouped into basal. The mutation rate in TCGA and the cell line panel shows a similar distribution across the subtypes. **Figure S3:** comparison of the best LS-SVM and RF models for the 90 compounds, sorted according to highest AUC obtained with either model. **Figure S4:** validation of the cell line signature for vorinostat in tumor samples grown in three dimensions: heatmap of the 150-gene signature for vorinostat in the cell line panel and 13 tumor samples treated with valproic acid. Seven out of eight sensitive samples (87.5%) and four out of five resistant samples (80%) are classified correctly with a probability threshold of 0.5 for response dichotomization. **Figure S5:** predicted probability of response of TCGA tumor samples to compounds lapatinib, sigma AKT1-2 inhibitor, GSK2126458 and docetaxel. The TCGA tumor samples are ordered according to increasing probability of response. **Figure S6:** correlation-based coherence heatmap for two cell line-derived gene signatures: coherence among 67 genes of the U133A signature for the sigma AKT1-2 inhibitor in the cell lines (left) and TCGA tumor samples (right) (Jaccard coefficient = 0.85; *P*-value <0.0001); coherence among 109 genes of the RNAseq signature for everolimus in the cell lines (left) and TCGA tumor samples (right) (Jaccard coefficient = 0.79; *P*-value <0.0001). **Figure S7:** comparison of the best model per dataset for the 90 compounds, sorted according to highest AUC obtained with either model (LS-SVM or RF). For RNAseq and exon array, the highest AUC is shown among models built on gene-level data only or all features (exons, junctions, and so on). **Figure S8:** distributions of response probabilities for 5-FU determined by mixed model clustering and used for cutoff selection. With a cutoff of 0.74, 23.9% of TCGA tumor samples were predicted to respond to 5-FU (Table S10 in Additional file 3). **Figure S9:** association between response to lapatinib and ERBB2 status, response to BIBW2992 and ERBB2 status, and response to tamoxifen and ER status for 306 TCGA patients with expression, methylation and copy number data available. **Figure S10:** heatmap of the 167 highest ranked features for lapatinib, obtained with RF applied to the full set of molecular data. Click here for file

Additional file 2: Table S1 Overview of 84 cell lines with subtype information and available data. GI_50_ values for 90 therapeutic compounds are provided for 70/84 cell lines included in all analyses. Click here for file

Additional file 3: Table S2 Processed Reverse Protein Lysate Array (RPPA) intensity data for 70 (phospho)proteins with fully validated antibodies in 49 cell lines. See Supplementary Methods in Additional file 3 for data processing details. Click here for file

Additional file 4: Table S3 GI_50_ dichotomization threshold for each compound, defined as the mean GI_50_ for the 48 core cell lines. Click here for file

Additional file 5: Table S5 Overview of the best LS-SVM/RF model for all 90 therapeutic compounds with comparison to the LS-SVM AUC based on subtype and ERBB2 status. For the subset of 51 therapeutic compounds with test AUC exceeding 0.7, additional information is provided on clinical trial status, comparison of GI_50_ with TGI, validation results of the cell line signal in the TCGA tumor samples, and most significant non-subtype related KEGG/BioCarta pathways from Additional file 6. Click here for file

Additional file 6: Table S7 List of significant non-subtype specific GO categories and KEGG/BioCarta pathways with FDR *P*-value <0.05. Per category/pathway information includes FDR *P*-value and the number of signature genes, percentage of signature genes and list of signature genes that are part of this category/pathway. Significant pathways associated with both drug response and transcriptional subtype were excluded, to capture biology underlying each compound’s mechanism of action. Click here for file

Additional file 7: Table S11 Compound response signatures for the 22 compounds featured in Figure 5 with model AUC >0.7 and at least one patient from the TCGA set of 306 tumor samples with expression, copy number and methylation data available with probability of response >0.65. Click here for file

Additional file 8: Table S14 Validation results for six drugs (BIBW2992, lapatinib, rapamycin, GSK2126458, gefitinib and GSK2141795) in 11 HER2+ lines. Click here for file

Additional file 9 **Raw drug response data.** Raw drug response data used to compute GI50 values used in this study. The columns represent the following: **cellline** = cell line lineage; **compound** = compound tested; **drug_plate_id** = unique identifier for the plate of 3 compounds; **T0_plate_id** = unique identifier for the time 0 h control plate associated with the drug plate; **background_od1, background_od2** = background od values (for correction of background luminesence); **od0.1, od0.2, od0.3** = triplicate measures for untreated cells; **od1.1, od1.2, od1.3… od9.1, od9.2, od9.3** = triplicate measures of number of cells alive after treatment with lowest to highest drug; **T0_background_od1, T0_background_od2** = background od values (for correction of background luminesence); **T0_median_od** = median od at T0; **c1 to c9** = drug concentrations tested; **units** = units of drug concentration tested. Click here for file
