# Erratum to: Modeling precision treatment of breast cancer

**DOI:** 10.1186/s13059-015-0658-5

**Published:** 2015-05-12

**Authors:** Anneleen Daemen, Obi L Griffith, Laura M Heiser, Nicholas J Wang, Oana M Enache, Zachary Sanborn, Francois Pepin, Steffen Durinck, James E Korkola, Malachi Griffith, Joe S Hur, Nam Huh, Jongsuk Chung, Leslie Cope, Mary Jo Fackler, Christopher Umbricht, Saraswati Sukumar, Pankaj Seth, Vikas P Sukhatme, Lakshmi R Jakkula, Yiling Lu, Gordon B Mills, Raymond J Cho, Eric A Collisson, Laura J van’t Veer, Paul T Spellman, Joe W Gray

**Affiliations:** Department of Cancer & DNA Damage Responses, Life Sciences Division, Lawrence Berkeley National Laboratory, Berkeley, CA 94720 USA; Laboratory Medicine, University of California San Francisco, San Francisco, CA 94115 USA; Department of Molecular and Medical Genetics, Oregon Health and Science University, Portland, OR 97239 USA; Department of Biomedical Engineering, Center for Spatial Systems Biomedicine, Knight Cancer Institute, Oregon Health and Science University, Portland, OR 97239 USA; Five3 genomics, 101 Cooper St, Santa Cruz, CA 95060 USA; The Genome Institute, Washington University School of Medicine, St Louis, MO 63105 USA; Samsung Electronics Headquarters, Seocho-gu, Seoul 137-857 Korea; Emerging Technology Research Center, Samsung Advanced Institute of Technology, Kyunggi-do, 446-712 Korea; Department of Oncology, Johns Hopkins University School of Medicine, Baltimore, MD 21205 USA; Department of Medicine, Beth Israel Deaconess Medical Center, Harvard Medical School, Boston, MA 02215 USA; Department of Systems Biology, MD Anderson Cancer Center, Houston, TX 77030 USA; Department of Dermatology, University of California, San Francisco, CA 94115 USA; Present address: Department of Bioinformatics & Computational Biology, Genentech Inc, 1 DNA Way, South San Francisco, CA 94080 USA; Present address: Sequenta Inc, South San Francisco, CA 94080 USA

## Abstract

During the type-setting of the final version of the article [1] some of the additional files were swapped. The correct files are republished in this Erratum.

The online version of the original article can be found under doi:10.1186/s13059-015-0658-5.

## Erratum

During the type-setting of the final version of the article [1] some of the additional files were swapped (with the legends remaining correct):

- Additional File 1 was published under Additional File 3 hyperlink;
- Additional File 2 was published under Additional File 1 hyperlink;
- Additional File 3 was published under Additional File 2 hyperlink.
- Additional Files 4–9 are all correct. The editors apologize for the clerical mistake that led to the mismatch of the additional files.

Additionally, the authors, based on the input from the readers, wish to expand some of the descriptions of the experimental procedures in the Additional File 1. In the section describing the mutation status analysis the authors added the following:

“For the alignment, pairs of Fastq files (i.e. R1 & R2) sequenced from the same sample were aligned separately using bwa aln & bwa sampe (default parameters) to the hg19 (GRCh37) reference. Each pair of Fastq files generates a single BAM file. Individual BAM files from the same sample were merged to generate a single BAM file representing all reads from the sequencing run. Using the GATK routine *CountCovariates*, the merged BAM file was subsequently analyzed to generate the covariates necessary to perform base quality recalibration. Briefly, it searches for mismatching bases in reads that do not overlap known heterozygous sites (1,000 genomes + dbSNP) and collects information on the mismatching base’s quality and a series of other covariates (e.g. base quality, read group, neighboring bases, sequencing cycle). Using the GATK routine *TableRecalibration*, the recalibration metrics obtained from *CountCovariates* were used to recalibrate all base qualities from the BAM file. This step is necessary as the base qualities generated by the sequencer often inaccurately reflect the true frequency of mismatching bases. The BAM files with base quality recalibration are the files used in all post-processing steps.

For mutation calling, allele counts and their associated base qualities were collected for each individual cell line. Only alleles fulfilling the following criteria were used in subsequent steps: base quality (**BQ**) > = 10; neighborhood base quality (**NBQ**) > = 10; mapping quality of associated read (**MQ**) > = 20; and its associated read is not a duplicate. Any base quality exceeding the read’s mapping quality is reduced to the read’s mapping quality. Positions with less than 2 reads supporting any non-reference allele were deemed homozygous reference and excluded from further analyses. The likelihoods of all possible genotypes (AA, AT, AC, etc.) given the allelic data collected for the cell line were computed using the MAQ error model originally defined in (*11*) and now available in the samtools source code. The genotype likelihoods were then used in a Bayesian model incorporating a prior probability on the reference, and the heterozygous rate of the human genome. The genotype with the highest likelihood given the data was chosen as most likely. No further analysis was performed at this position for a homozygous reference genotype. Otherwise, the following metrics were computed at the variant position and used for post-processing filtering of all putative variants: **DP**: Total read depth, **AD**: Depth or coverage for all alleles, including alleles not in genotype; **BQ**: Average base quality of each allele; **MQ**: Average mapping quality of reads supporting each allele**; MQ0**: Number of mapping quality zero reads overlapping position; **MQL**: Number of ‘low’ mapping quality reads overlapping position; **NAHP**: Average number of adjacent homopolymer runs on either side of each allele in genotype; **MAHP**: Longest adjacent homopolymer run on either side of each allele in genotype; **AMM**: Average number of mismatches in reads supporting each allele; **MMQS**: Average sum of the base qualities for all mismatching bases**; DETP**: Average effective distance to 3’ end of read for each allele, normalized by read length**; LD/MD/RD**: Number of reads supporting each allele where the allele is located in the left-most third of read, middle-third of read, or right-most third of read, respectively**; LDS/MDS/RDS**: Strand-aware version of above**; SB**: Number of reads supporting each allele aligned to the forward strand; and **PN/NN**: Previous and next nucleotides in reference.

Since no normal control is available for our cell lines, all variants were considered germline and the genotype’s log-likelihood was used to compute a Phred-scaled quality/confidence of the germline variant. All putative variants and associated metrics were converted to the VCF format, with the following filters applied to each variant: **conf**: Genotype quality > = 100; **dp**: Total depth > = 8; **mdp**: Maximum depth < 800; **mq0**: MQ0 < 5; **mql**: MQL < 5; **sb**: Mutant allele strand bias p-value > 0.005 (Binomial test); **mmqs**: MMQS < = 20; **amm**: AMM < = 1.5; **detp**: 0.2 < = DETP < = 0.8; **ad**: AD of mutant allele > = 4; and **ma**: More than two alleles have read support > = 2. Variants that pass all filters were marked PASS in the FILTER column of their VCF record. Otherwise, the names of each filter that the variant does not meet were recorded in the FILTER column.

Read coverage was calculated using a dynamic windowing approach that expands and contracts the window’s genomic width according to the local read density in the sample’s sequence. When the window’s read count exceeds a user-defined threshold, the window’s size and location, the raw read count, *N*, and the average coverage of the window, *N* / window size, were recorded.”

The correct Additional files 1, 2 and 3, which include the expanded description of the methods, are published below.

## Additional files

Additional file 1:
**Supplementary Methods, Supplementary Results, Figures S1 to S10, and Tables S4, S6, S8, S9, S10, S12, and S13. Supplementary Methods:** detailed description of the therapeutic compound response data, molecular data for the breast cancer cell lines, molecular data for the external breast cancer tumor samples used for validation, classification methods, data integration approach, statistical methods, and pathway overrepresentation analysis. **Supplementary Results:** assessment of cell line signal in tumor samples, inter-data relationships, prediction comparison of datasets, validation against other cell line datasets, and the patient response prediction toolbox for the R project for statistical computing. **Table S4:** overview of genes with good correlation (FDR *P*-value <0.05) between SNP6 and gene expression; 22 to 39% of genes in copy number aberration regions show a significant concordance between their genomic and transcriptomic profile after multiple testing correction. **Table S6:** data type ranking of the importance of the molecular datasets by comparison of prediction performance of LS-SVM and RF classifiers built on individual data sets and their combination, and by comparison of the average appearance of data types in the top 100 of ranked features, with and without inclusion of RPPA data. Examples are also provided of compounds for which (most) datasets give similar results or for which one dataset performs better (shown in bold). **Table S8:** performance for 'splice-specific' response predictors (RF) with an AUC increase >0.05 when comparing all transcript features to gene-level values alone. **Table S9:** statistical association between clinical variables and predicted response for 306 TCGA patients with expression, methylation and copy number data available. For each compound, the best performing model was utilized (LS-SVM or RF with any combination of expression, copy number and methylation data). **Table S10:** resistant/intermediate/sensitive cutoffs for 22 compounds with model AUC >0.7 and at least one patient with probability of response >0.65. Cutoff value 1 separates patients considered resistant from intermediate. Cutoff value 2 separates patients considered intermediate from sensitive. The percentage value for each group indicates the percentage of total patients (n = 306) in each group. **Table S12:** presence and variance of filtered features from U133A and exon array cell line data in tumor samples. Features from U133A and the exon array that passed the variance and presence filter in the cell lines were present in the majority of breast cancer tumor samples. **Table S13:** summary of 167 predictors in random forests classifier for lapatinib (all data types, optimal predictor number). **Figure S1:** data summary in terms of number of features before and after data-type-specific reduction and unsupervised filtering based on variance and signal detection above background. **Figure S2:** overview of the mutation prevalence in the cell line panel and TCGA data set for the list of seven common coding variants detected by TCGA, with a distinction between luminal, basal and ERBB2-enriched. Cell lines with unknown subtype are displayed in orange. To make the subtypes comparable, luminal A and B were grouped into luminal for the TCGA data set, whilst basal and claudin-low cell lines were grouped into basal. The mutation rate in TCGA and the cell line panel shows a similar distribution across the subtypes. **Figure S3:** comparison of the best LS-SVM and RF models for the 90 compounds, sorted according to highest AUC obtained with either model. **Figure S4:** validation of the cell line signature for vorinostat in tumor samples grown in three dimensions: heatmap of the 150-gene signature for vorinostat in the cell line panel and 13 tumor samples treated with valproic acid. Seven out of eight sensitive samples (87.5%) and four out of five resistant samples (80%) are classified correctly with a probability threshold of 0.5 for response dichotomization. **Figure S5:** predicted probability of response of TCGA tumor samples to compounds lapatinib, sigma AKT1-2 inhibitor, GSK2126458 and docetaxel. The TCGA tumor samples are ordered according to increasing probability of response. **Figure S6:** correlation-based coherence heatmap for two cell line-derived gene signatures: coherence among 67 genes of the U133A signature for the sigma AKT1-2 inhibitor in the cell lines (left) and TCGA tumor samples (right) (Jaccard coefficient = 0.85; *P*-value <0.0001); coherence among 109 genes of the RNAseq signature for everolimus in the cell lines (left) and TCGA tumor samples (right) (Jaccard coefficient = 0.79; *P*-value <0.0001). **Figure S7:** comparison of the best model per dataset for the 90 compounds, sorted according to highest AUC obtained with either model (LS-SVM or RF). For RNAseq and exon array, the highest AUC is shown among models built on gene-level data only or all features (exons, junctions, and so on). **Figure S8:** distributions of response probabilities for 5-FU determined by mixed model clustering and used for cutoff selection. With a cutoff of 0.74, 23.9% of TCGA tumor samples were predicted to respond to 5-FU (Table S10 in Additional file 1). **Figure S9:** association between response to lapatinib and ERBB2 status, response to BIBW2992 and ERBB2 status, and response to tamoxifen and ER status for 306 TCGA patients with expression, methylation and copy number data available. **Figure S10:** heatmap of the 167 highest ranked features for lapatinib, obtained with RF applied to the full set of molecular data. Additional file 2:
**Table S1.** Overview of 84 cell lines with subtype information and available data. GI_50_ values for 90 therapeutic compounds are provided for 70/84 cell lines included in all analyses. Additional file 3:
**Table S2.** Processed Reverse Protein Lysate Array (RPPA) intensity data for 70 (phospho)proteins with fully validated antibodies in 49 cell lines. See Supplementary Methods in Additional file 1 for data processing details.

## References

[1] Daemen A, Griffith OL, Heiser LM, Wang NJ, Enache OM, Sanborn Z (2013). Modeling precision treatment of breast cancer. Genome Biol.

