# Supplementary material for: Erratum to: Modeling precision treatment of breast cancer
Source: Genome Biol. 2015 May 12;16(1):95. doi: 10.1186/s13059-015-0658-5 (PMC4426644; doi:10.1186/s13059-015-0658-5)
Supplement: Additional file 1: — Supplementary Methods, Supplementary Results, Figures S1 to S10, and Tables S4, S6, S8, S9, S10, S12, and S13. Supplementary Methods: detailed description of the therapeutic compound response data, molecular data for the breast cancer cell lines, molecular data for the external breast cancer tumor samples used for validation, classification methods, data integration approach, statistical methods, and pathway overrepresentation analysis. Supplementary Results: assessment of cell line signal in tumor samples, inter-data relationships, prediction comparison of datasets, validation against other cell line datasets, and the patient response prediction toolbox for the R project for statistical computing. Table S4: overview of genes with good correlation (FDR P-value <0.05) between SNP6 and gene expression; 22 to 39% of genes in copy number aberration regions show a significant concordance between their genomic and transcriptomic profile after multiple testing correction. Table S6: data type ranking of the importance of the molecular datasets by comparison of prediction performance of LS-SVM and RF classifiers built on individual data sets and their combination, and by comparison of the average appearance of data types in the top 100 of ranked features, with and without inclusion of RPPA data. Examples are also provided of compounds for which (most) datasets give similar results or for which one dataset performs better (shown in bold). Table S8: performance for 'splice-specific' response predictors (RF) with an AUC increase >0.05 when comparing all transcript features to gene-level values alone. Table S9: statistical association between clinical variables and predicted response for 306 TCGA patients with expression, methylation and copy number data available. For each compound, the best performing model was utilized (LS-SVM or RF with any combination of expression, copy number and methylation data). Table S10: resistant/intermediate/sensitive cutoffs for 22 compounds with [file 13059_2015_658_MOESM1_ESM.docx]

**Modeling precision treatment of breast cancer**

**Authors:** Anneleen Daemen^§,*^, Obi L Griffith^§,*^, Laura M Heiser^^^, Nicholas J Wang^^^, Oana M Enache, Zachary Sanborn, Francois Pepin, Steffen Durinck, James E Korkola_,_ Malachi Griffith, Joe S Hur, Nam Huh, Jongsuk Chung, Leslie Cope, Mary J Fackler, Christopher Umbricht, Saraswati Sukumar, Pankaj Seth, Vikas P Sukhatme, Lakshmi R Jakkula, Yiling Lu, Gordon B. Mills, Raymond J Cho, Eric A Collisson, Laura J van’t Veer, Paul T Spellman, Joe W Gray^*^

*To whom correspondence should be addressed: [daemena@gene.com](mailto:daemena@gene.com), [ogriffit@genome.wustl.edu](mailto:ogriffit@genome.wustl.edu), [grayjo@ohsu.edu](mailto:grayjo@ohsu.edu).

^§^These authors contributed equally to this work

^These authors contributed equally to this work

**Supplementary Methods:**

*Therapeutic compound response data* As previously described ([*1*](#_ENREF_1)), cells were treated for 72 hours with a set of 9 doses of each compound in 1:5 serial dilution. Nonlinear least squares was used to fit the data with a Gompertz curve, to obtain the compound concentration required to inhibit growth by 50% (GI_50_) and the concentration that elicited total growth inhibition (TGI). Based on the GI_50_ values, compounds were excluded from the analyses when GI_50_ was missing in more than 40% of the entire set of cell lines, or when the log10-transformed GI_50_ value exceeded 1 standard deviation below or above the average log10-transformed GI_50_ in less than five cell lines. Based on these rules, 48 compounds were dropped for various reasons. These compounds are not listed here. Because of the various reasons, dropping does not imply improper functioning of the compounds.

*Molecular data of breast cancer cell lines*

*Copy number (SNP6):* DNA extracted from cell lines was labeled and hybridized to the Affymetrix Genome-Wide Human SNP Array 6.0 in two batches (2007 and 2009) for DNA copy number. The determination of array quality and data processing was performed using the 'aroma.affymetrix' package in R ([*2*](#_ENREF_2)). Data were normalized as previously described ([*3*](#_ENREF_3)) and DNA copy number ratios at each locus were estimated relative to a set of 20 normal sample arrays. Data were segmented using the circular binary segmentation (CBS) algorithm from the Bioconductor package DNAcopy ([*4*](#_ENREF_4)), followed by summarization at gene level with the R package CNTools. Missing values were imputed with KNNimputer in R ([*5*](#_ENREF_5)) and genes with missing values in >50% of cell lines were excluded (only 5). We used genome build HG18 for processing and annotating. Raw data for the 2007 batch are available in The European Genotype Archive (EGA) with accession number, EGAS00000000059, and were published in conjunction with Heiser, et al 2011 ([*1*](#_ENREF_1)). Raw data for the 2009 batch are available in EGA with accession number, EGAS00001000585*.* The summarized copy number ratios at gene level after missing value imputation were used as molecular features in all analyses.

*Gene expression:* Gene expression data for the cell lines were derived from Affymetrix GeneChip Human Genome U133A and Affymetrix GeneChip Human Exon 1.0 ST arrays. The U133A expression data were preprocessed with the RMA algorithm in R. The maximum varying probe set per gene was selected, reducing the set of 22,283 probe sets to 12,789 unique genes. The raw data for expression profiling are available at ArrayExpress (<http://www.ebi.ac.uk/arrayexpress/>) with accession number E-TABM-157. RMA processed gene expression at log2 scale were used as molecular features in all analyses. For the validation of expression-based predictors of sensitivity to the HDAC inhibitors and tamoxifen, both with case-control expression data with outcome available for tumor samples, the best performing predictor out of the 4 following preprocessing strategies was used: preprocessing of U133A expression data with the RMA or GCRMA algorithm in R, and summarization with Affymetrix standard annotation or a customized CDF annotation file ([*6*](#_ENREF_6)). For the exon array data, gene-level summaries of expression were computed using 'aroma.affymetrix' in R ([*2*](#_ENREF_2)) with quantile normalization and a log-additive probe-level model (PLM) (default settings). An improved mapping of the probes to human genome build 36.1 obtained by TCGA was used ([*7*](#_ENREF_7)). Transcript and exon level summaries were computed following the same approach, but with use of the annotation file for the HuEx-1_0-st-v2 chip type, restricted to the core probe sets according to version R3. The raw data are available in ArrayExpress (E-MTAB-181). Gene-level summaries were used as molecular features for the exon array models at gene level. Exon array models with all features include summaries at gene, transcript and exon level.

*Transcriptome sequencing:* Whole transcriptome shotgun sequencing (RNAseq) was completed on breast cancer cell lines. RNAseq libraries were prepared using the TruSeq RNA Sample Preparation Kit (Illumina) and Agilent Automation NGS system per manufacturers’ instructions. Sample prep began with 1 μg of total RNA from each sample. Poly-A RNA was purified from the sample with oligo dT magnetic beads, and the poly(A) RNA was fragmented with divalent cations. Fragmented poly-A RNA was converted into cDNA through reverse transcription and were repaired using T4 DNA polymerase, Klenow polymerase, and T4 polynucleotide kinase. 3’ A-tailing with exo-minus Klenow polymerase was followed by ligation of Illumina paired-end oligo adapters to the cDNA fragment. Ligated DNA was PCR amplified for 15 cycles and purified using AMPure XP beads. After purification of the PCR products with AMPure XP beads, the quality and quantity of the resulting libraries were analyzed using an Agilent Bioanalyzer High Sensitivity chip.

Expression analysis was performed with the ALEXA-seq software package as previously described ([*8*](#_ENREF_8)). Briefly, this approach comprises (i) creation of a database of expression and alternative expression sequence ‘features’ (genes, transcripts, exons, junctions, boundaries, introns, and intergenic sequences) based on Ensembl gene models, (ii) mapping of short paired-end sequence reads to these features by BLAST and BWA, (iii) identification of features that are expressed above background noise while taking into account locus-by-locus noise. RNAseq data was available for 56 lines. An average of 71.1 million (76bp paired-end) reads passed quality control per sample. Of these, 53.9 million reads mapped to the transcriptome on average, resulting in an average gene depth of coverage of 48.4X (i.e., average number of alignments per bp across all genes). Log2 transformed estimates of gene-level expression were extracted for inclusion as molecular features in the analyses with corresponding expression status values indicating whether the genes were detected above background level. RNAseq models with all features include all sequence features, expressed above background noise. Sequencing data is available at Gene Expression Omnibus (<http://www.ncbi.nlm.nih.gov/geo/>) with accession number GSE48216.

*Genome-wide methylation assay:* The Illumina Infinium Human Methylation27 BeadChip Kit was used for the genome-wide detection of 27,578 CpG loci, spanning 14,495 genes ([*9*](#_ENREF_9)). Bisulfite-converted DNA was analyzed in the DNA Microarray Core, Johns Hopkins University (Sukumar lab). Using the GenomeStudio Methylation Module v1.0 software, methylation for each CpG locus was expressed as a beta value according to the formula: beta = (signal intensity of M probe) / [(signal intensity of M + U probes) + 100]. The M and U probes allow measurement of the abundance of methylated and unmethylated DNA, respectively, at each single CpG locus. Hence, beta values range from 0 (completely unmethylated) to 1 (completely methylated), and are proportional to the degree of methylation at any particular locus. Cell lines were characterized by focal hypermethylation and global hypomethylation. Before filtering, on average 46.5% of 27,578 CpG loci had a beta value below 0.1, 57.1% below 0.2, and the beta values of on average 19.6% of CpG loci exceeded 0.8. When focusing on the 7,424 CpG loci after prefiltering, the percentages for hypomethylation increased to 60.1% and 74.3%, and hypermethylation occurred on average for 6.2% of loci. Genome-wide methylation beta values are available at Gene Expression Omnibus (<http://www.ncbi.nlm.nih.gov/geo/>) with accession number GSE42944. Beta values for all CpG loci were used as molecular features in all analyses.

*Protein abundance (RPPA):* Reverse protein lysate array (RPPA) is an antibody-based method to quantitatively measure protein abundance ([*10*](#_ENREF_10)). RPPA data were generated and pre-processed by the Gordon Mills lab at MD Anderson. Cells were exponentially grown prior to harvesting and not subject to any particular treatment. The intensity in 49 cell lines was measured for 146 (phospho)proteins, with many proteins represented by multiple isoforms (e.g., native and phosphorylated forms). Of these 146 proteins, 70 (48%) proteins with fully validated antibody were included in the analysis. Data included in all analyses are available in **Table S2**.

*Mutation status*: Mutation status was obtained from exome-capture sequencing (see Cho RJ et al, in preparation). Exome libraries of tumor and some normal genomic DNAs were generated using the Agilent SureSelect XT kit and Agilent Automation Systems NGS system per manufacturer’s instructions. 1 ug of each genomic DNA was sheared using a Covaris E220 to a peak target size of 150 bp. Fragmented DNA was concentrated using AMPureXP beads (Beckman Coulter), and DNA ends were repaired using T4 DNA polymerase, Klenow polymerase, and T4 polynucleotide kinase. 3’ A-tailing with exo-minus Klenow polymerase was followed by ligation of Agilent paired-end oligo adapters to the genomic DNA fragment. Ligated DNA was PCR amplified for 8 cycles and purified using AMPure XP beads and quantitated using the Quant-It BR kit (Invitrogen). 500 ng of sample libraries were hybridized to the Agilent biotinylated SureSelect 37Mb Capture Library at 65°C for 72 hr following the manuufacturer’s protocol. The targeted exon fragments were captured on Dynabeads MyOne Strepavidin T1 (Invitrogen), washed, eluted, and enriched by amplification with Agilent post-capture primer and an indexed reverse primer for multiplexing12 additional cycles. After purification of the PCR products with AMPure XP beads, the quality and quantity of the resulting exome libraries were analyzed using an Agilent Bioanalyzer High Sensitivity chip.

For the alignment, pairs of Fastq files (i.e. R1 & R2) sequenced from the same sample were aligned separately using bwa aln & bwa sampe (default parameters) to the hg19 (GRCh37) reference. Each pair of Fastq files generates a single BAM file. Individual BAM files from the same sample were merged to generate a single BAM file representing all reads from the sequencing run. Using the GATK routine *CountCovariates*, the merged BAM file was subsequently analyzed to generate the covariates necessary to perform base quality recalibration. Briefly, it searches for mismatching bases in reads that do not overlap known heterozygous sites (1,000 genomes + dbSNP) and collects information on the mismatching base’s quality and a series of other covariates (e.g. base quality, read group, neighboring bases, sequencing cycle). Using the GATK routine *TableRecalibration*, the recalibration metrics obtained from *CountCovariates* were used to recalibrate all base qualities from the BAM file. This step is necessary as the base qualities generated by the sequencer often inaccurately reflect the true frequency of mismatching bases. The BAM files with base quality recalibration are the files used in all post-processing steps.

For mutation calling, allele counts and their associated base qualities were collected for each individual cell line. Only alleles fulfilling the following criteria were used in subsequent steps: base quality (**BQ**) >= 10; neighborhood base quality (**NBQ**) >= 10; mapping quality of associated read (**MQ**) >= 20; and its associated read is not a duplicate. Any base quality exceeding the read’s mapping quality is reduced to the read’s mapping quality. Positions with less than 2 reads supporting any non-reference allele were deemed homozygous reference and excluded from further analyses. The likelihoods of all possible genotypes (AA, AT, AC, etc.) given the allelic data collected for the cell line were computed using the MAQ error model originally defined in ([*11*](#_ENREF_11)) and now available in the samtools source code. The genotype likelihoods were then used in a Bayesian model incorporating a prior probability on the reference, and the heterozygous rate of the human genome. The genotype with the highest likelihood given the data was chosen as most likely. No further analysis was performed at this position for a homozygous reference genotype. Otherwise, the following metrics were computed at the variant position and used for post-processing filtering of all putative variants: **DP**: Total read depth, **AD**: Depth or coverage for all alleles, including alleles not in genotype; **BQ**: Average base quality of each allele; **MQ**: Average mapping quality of reads supporting each allele**; MQ0**: Number of mapping quality zero reads overlapping position; **MQL**: Number of ‘low’ mapping quality reads overlapping position; **NAHP**: Average number of adjacent homopolymer runs on either side of each allele in genotype; **MAHP**: Longest adjacent homopolymer run on either side of each allele in genotype; **AMM**: Average number of mismatches in reads supporting each allele; **MMQS**: Average sum of the base qualities for all mismatching bases**; DETP**: Average effective distance to 3’ end of read for each allele, normalized by read length**; LD/MD/RD**: Number of reads supporting each allele where the allele is located in the left-most third of read, middle-third of read, or right-most third of read, respectively**; LDS/MDS/RDS**: Strand-aware version of above**; SB**: Number of reads supporting each allele aligned to the forward strand; and **PN/NN**: Previous and next nucleotides in reference.

Since no normal control is available for our cell lines, all variants were considered germline and the genotype’s log-likelihood was used to compute a Phred-scaled quality / confidence of the germline variant. All putative variants and associated metrics were converted to the VCF format, with the following filters applied to each variant: **conf**: Genotype quality >= 100; **dp**: Total depth >= 8; **mdp**: Maximum depth < 800; **mq0**: MQ0 < 5; **mql**: MQL < 5; **sb**: Mutant allele strand bias p-value > 0.005 (Binomial test); **mmqs**: MMQS <= 20; **amm**: AMM <= 1.5; **detp**: 0.2 <= DETP <= 0.8; **ad**: AD of mutant allele >= 4; and **ma**: More than two alleles have read support >= 2. Variants that pass all filters were marked PASS in the FILTER column of their VCF record. Otherwise, the names of each filter that the variant does not meet were recorded in the FILTER column.

Read coverage was calculated using a dynamic windowing approach that expands and contracts the window’s genomic width according to the local read density in the sample’s sequence. When the window’s read count exceeds a user-defined threshold, the window’s size and location, the raw read count, *N*, and the average coverage of the window, *N* / window size, were recorded.

Finally for variant filtering, mutations across all cell lines were filtered based on following criteria: 1) average sum of the base quality scores of all mismatches in the reads containing the mutant allele ≤ 20; 2) average number of other mismatches in the reads ≤ 1.5; 3) average distance of the mutant alleles to the 3’ end of their respective reads between 0.2 and 0.8; 4) mutant allele read support ≥ 4; 5) number of reads per variant supporting either the reference or mutant allele < 400; 6) mutation not present in dbSNP build 131. Sequencing data is available at Gene Expression Omnibus (<http://www.ncbi.nlm.nih.gov/geo/>) with accession number GSE48216.

In addition to the data-type-specific reduction of the number of features, unsupervised filters based on variance and signal detection above background were applied to the data sets where applicable ([*12*](#_ENREF_12)). For the methylation data, CpG loci that varied most across all cell lines were incorporated, requiring the coefficient of variance (COV), defined as the ratio of the standard deviation to the mean, to be within the range of 0.7 to 10. For the expression and transcriptome sequence data sets, a presence filter was applied in addition to the variance filter. In at least 20% of the cell lines the raw expression level was required to exceed 100 for U133A probe sets, 100 for the gene- and transcript-level summaries from the exon array, 150 for the exon-level summaries from the exon array, and a gene-specific level obtained by ALEXA-seq for RNAseq. To make the filters comparable across data types, the lower limit on COV was increased from 0.7 to 1.05 for the exon-level summaries from the exon array and to 2 for RNAseq. For the SNP6 data, DNA copy number ratios were filtered based on standard deviation instead of COV, with use of the same lower limit of 0.7.

*Molecular data of breast cancer tumor samples* The following data sets were used to verify whether features obtained from the cell line data after filtering were detectable and varied in clinical tumor samples. For U133A 672 samples from 9 different studies were included, being a mix of normal-like, luminal, basal, inflammatory and locally advanced breast carcinoma samples of different stage ([*13*](#_ENREF_13)). The RMA-normalized data were downloaded from [http://www.ebi.ac.uk/gxa/array/U133A](http://www.ebi.ac.uk/gxa/array/U133A%29). Exon array data were available for 84 tumor samples, with a similar number of ERBB2-positive, luminal and basal tumors (GSE16534) ([*14*](#_ENREF_14)). In The Cancer Genome Atlas (TCGA) project, methylation data were collected (as of June 2011) for 183 breast tumor samples (downloaded from <http://tcga-data.nci.nih.gov/tcga/>). The exon array and methylation data were preprocessed in the same manner as for the cell line data.

For both U133A and exon array, distributions of tumor and cell line expression data were highly similar. However, before determining the percentage of tumor samples per gene for which expression exceeded background level, this level was adapted such that the difference between background level and the median of the distribution of all features was the same in the cell line panel and in both tumor data sets, resulting in a tumor-specific background level of 195 for U133A and 70 for the exon array.

The tumor set considered for prevalence and patient population characterization consisted of 536 breast invasive carcinoma samples collected by TCGA as of February 10, 2012, with focus on ductal and lobular carcinoma. Gene expression (AgilentG4502A), copy number (Affymetrix Genome-Wide Human SNP Array 6.0) and methylation data (Illumina Infinium Human Methylation27 Beadchip Kit) have been collected for 536, 523 and 318 of these samples, respectively. Missing values in these data sets were imputed with KNNimputer in R([*5*](#_ENREF_5)). To verify whether a compound’s subtype specificity in the cell line panel was similar to that seen in the tumor samples, the TCGA samples were assigned the subtype luminal, basal, claudin-low, normal-like, or ERBB2-amplified using PAM50 ([*15*](#_ENREF_15)). No distinction was made between luminal A and B samples in accordance with the cell line data.

Because a 3D culture microenvironment more closely resembles the *in vivo* microenvironment compared to 2D cultures ([*16*](#_ENREF_16)) ([*17*](#_ENREF_17)*,* [*18*](#_ENREF_18)), we investigated whether signatures derived from 2D response data perform well in 3D culture. In a study on valproic acid, a histone deacetylase (HDAC) inhibitor, patient breast tumor samples both from primary tumors and pleural effusions were grown in 3-dimensional *in vitro* conditions and expression data were collected with the Affymetrix U133 plus 2 platform ([*19*](#_ENREF_19)). A dose-response assay was carried out to determine the effective concentration at which growth rate is inhibited by 50% (EC50). Growth inhibition for 13 samples ranged from 3.9 to 873.8 nM. Eight samples with similar EC50 below 12 were called sensitive, whilst the remaining 5 samples with EC50 of 27 or higher were called resistant. Five HDAC inhibitors were included in our panel of compounds, being valproic acid, vorinostat, trichostatin A, LBH589 and oxamflatin. The GI_50_ values of those compounds all passed the minimum variation filter in our cell line panel([*1*](#_ENREF_1)). Based on the U133A expression data, optimal response prediction result in the cell lines was obtained with the LS-SVM classifier for the compound vorinostat, with an average test AUC on our cell line panel of 0.77 when the data were preprocessed with GCRMA with use of a customized CDF annotation file ([*6*](#_ENREF_6)). This signature was validated on the 13 tumor samples grown in 3D with the appropriate preprocessing of the expression data and with inclusion of the highest ranked genes that had a p-value < 0.05 on average across the 100 training randomizations.

For validation of Tamoxifen sensitivity models, studies were collected which provided gene expression data for patients that received Tamoxifen therapy. Each study was required to have a sample size of at least 100 and report some measure of outcome (RFS = relapse free survival, DMFS = distant metastasis free survival, or DFS = disease free survival). To allow combination of the largest number of samples, the common Affymetrix U133A gene expression platform was used. Four studies ([*20-23*](#_ENREF_20)) meeting the above criteria were identified by searching the Gene Expression Omnibus (GEO) database ([*24*](#_ENREF_24)) corresponding to GEO dataset accessions: GSE6532, GSE12093, GSE17705, and GSE2990. After filtering for only those samples which were Tamoxifen-treated, removing duplicates and excluding patients with perioperative events, 439 patient samples remained. All data processing and analyses were completed with open source R/Bioconductor packages. Raw data (CEL-files) were downloaded from GEO. Duplicate samples were identified and removed if they had the same database identifier (e.g., GSM accession), same sample/patient id, or showed a high correlation (r > 0.99) compared to any other sample in the dataset. Raw data were normalized and summarized using the 'affy' and 'gcrma' libraries (that is, GCRMA preprocessing with Affymetrix standard annotation). Probes were mapped to Entrez gene symbols using standard annotation files. Classification models built on cell line data were then applied to this tamoxifen-treated dataset of 439 patients. Patients were categorized as either tamoxifen-sensitive or tamoxifen-resistant (response class). Kaplan-Meier survival analysis was performed using the R ‘survival’ package for relapse free survival with response class as the factor and p-value determined by log-rank test. Events beyond 10 years were censored.

*Classification methods* Two classification methods have been used throughout the manuscript, being the Least Squares Support Vector Machine (LS-SVM) and Random Forests. For the LS-SVM ([*25*](#_ENREF_25)) ([*26*](#_ENREF_26)), the cell line panel was randomly split into 2/3rd training and 1/3rd test, with the split stratified to outcome. The training set was used for the optimization of the regularization parameter of the LS-SVM and the number of features included in each classifier. Five-fold cross-validation (CV) was applied to the training set in combination with a grid search approach to determine the optimal parameter combination among 40 possible values for the regularization parameter and number of features, both on a logarithmic scale. The model, rebuilt on the training set with the optimal parameter setting, was subsequently validated on the left-out test samples, with performance reported as the area under the receiver operating characteristics (ROC) curve (AUC). This randomization strategy was repeated 100 times. Due to the large uncertainty in AUC estimates ([*27*](#_ENREF_27)), a two-sided 95% confidence interval for both test and train AUC were calculated as the average AUC +/- the standard error of the mean (SEM) multiplied by a t-factor. SEM is defined as the standard deviation divided by the square root of the number of cell lines. The t-factor is obtained from the t distribution based on a given degree of freedom (number of cell lines minus 1) and a two-sided significance level of 0.05. A t distribution was used over a normal distribution because the unknown variance on AUC had to be estimated from sample data. For each separate experiment described herein, the degree of freedom was set to the median degree of freedom across all compounds per data type or combination of data types. Biological focus is restricted to classifiers for which the 95% confidence interval does not contain AUC=0.5. Subsequently to take unbalanced class sizes into account, a weighted version of the LS-SVM was used ([*28*](#_ENREF_28)). As kernel function, the clinical kernel function was opted for ([*29*](#_ENREF_29)), which takes the range and type of each feature into account, thereby equalizing the influence of each feature on the cell line similarity matrix and thus classifier. The kernel function for variable *z* between samples *i* and *j* equals *kz(i,j) = (r - |zi-zj|) / r*, with *r* the range for continuous variables and the number of categories minus 1 for ordinal and binary variables. The global kernel function is the average of the kernel functions across all features. The range for the data types was based on the full data set, with r=10 for SNP6, r=11 for U133A, r=12 for gene and transcript summaries from the exon array, r=15 for exon summaries from the exon array, r=18 for RNAseq except for active introns and active intergenic regions with r=13 and 14, respectively, r=15 for RPPA, and r=1 for both methylation and mutation data. To transform the output of the LS-SVM into a predicted probability from 0 to 1, a sigmoid function with parameters A and B was trained on the cell line data ([*30*](#_ENREF_30)). Finally as feature selection methods, the Wilcoxon rank sum test was used for continuous variables, Kruskal Wallis test for ordinal variables, and Fisher’s exact test for nominal variables.

Random Forests classification was performed using the R 'randomForest' library with response as the target (as determined by mean GI_50_). Forests were created with at least 10,001 trees (odd number ensures fully deterministic model) and otherwise default settings. To compensate for unequal class sizes, random down-sampling of the larger class to the size of the smaller class was performed for each tree in the forest. The optimal number of predictor variables was estimated using the ‘rfcv’ function in the randomForest library. A total of 50 replicates were performed with five-fold CV and step of 0.9 on a log scale. The number of predictors with minimum mean CV error across the replicates was chosen as optimal predictor number. Random Forests models were saved for both the complete set of predictors and the optimal set. Performance was assessed by ROC AUC reported values for Random Forests internal out-of-bag (OOB) testing. The OOB testing is based on the fact that each tree in the forest is built on a random 2/3 subset of patients and the remaining 1/3 used as test set for that tree. For classifiers built on the combined dataset (all molecular data types) missing values were imputed with ‘rfImpute’ using subtype as the target.

*Comparison of classifiers* The Least Squares Support Vector Machine is the least squares modified version of the Support Vector Machine (SVM), a kernel method for supervised classification. Kernel methods are a group of algorithms that operate on the kernel matrix, a sample-by-sample similarity matrix with dimension determined by the number of samples, regardless of number and complexity of features. With supervised classification, the best possible hyperplane is determined among all hyperplanes that separate two classes of samples. The SVM classifier is obtained from the solution to a convex optimization problem in which the margin around the hyperplane is maximized, subject to the constraint of correctly classifying the training samples with strong confidence. For problems with high dimensional data, the problem is typically solved as a quadratic programming (QP) problem. Whilst a QP problem requires expensive computation, the least squares modifications transform the QP problem into a much simpler set of linear equations. The random forests classification method on the other hand involves creation of a collection of decisions trees. As each tree is grown, if there are M input variables, a smaller number m are randomly selected out of M and the best splitter from these is used to split the cases at that node into the target classes. The tree is extended until all cases are correctly classified. The value of m is held constant during the forest growing. Each tree is grown from a random 2/3 sampling (with replacement) of N cases in the training data. The 1/3 of cases not sampled are used in an internal out-of-bag (OOB) test of that tree. Overall performance (AUC, error rate, etc) is estimated from the sum of all OOB tests. When applied to a sample of unknown status, each tree in the forest gets one “vote” towards the predicted class of that sample.

The LS-SVM has several advantages including its ability to handle a wide range of data types and integrate heterogeneous data through the use of the kernel matrix, and its computational speed for high dimensional data. Another advantage is its classification robustness to uncertainties in the data through regularization between minimization of the classification error and maximization of the generalizability of the classifier to new data. Advantages of the random forests method include its ability to handle a large number of variables without a preliminary feature selection, estimates of variable importance, and internal OOB testing that produces accurate and unbiased estimates of performance without the need for separate computation or setting aside independent test data.

*Data integration* Classifiers were built on the molecular data sets separately as well as on the combined data. For the latter, missing molecular data for 47/48 core cell lines were imputed with Random Forests (‘rfimpute’), using subtype as target variable. Core cell line HBL100 was excluded due to its unknown subtype status. For data integration, an early integration strategy was used, concatenating all individual data sets before building the classifier ([*31*](#_ENREF_31)). Because the distributions of the molecular data sets are not comparable, the influence of each data set on classification was equalized for the LS-SVM by using a kernel function that accounts for variable range ([*29*](#_ENREF_29)). For Random Forests, we verified for lapatinib that differences in the dimensionality of the data sets do not drive the significant associations.

**Fig. S9** displays the 167 (of a total 44,473) highest ranked features for lapatinib, with mainly ERBB2-positive cell lines sensitive to this compound (from Random Forests, all data types). Of these, 132/167 (79.0%) of variables were from loci in the known ERBB2 amplicon region (*CRKRS*, *NEUROD2*, *PPP1R1B*, *STARD3*, *TCAP*, *PNMT*, *PERLD1*, *ERBB2*, *C17orf37*, *GRB7*, *ZNFN1A3*) (**Table S13**). ERBB2 amplicon genes were represented at the levels of copy number (SNP6), transcript expression (RNAseq, exon array, U133A), and protein expression (RPPA). The top ranking variables were dominated by SNP6 variables (including all 11 amplicon genes listed above), with the next most useful predictors coming from RNAseq (*GRB7*), exon array (*ERBB2*, *GRB7* and *PERLD1*), U133A (*GRB7*) and finally RPPA (ERBB2p1248). An additional 35 variables were included that did not belong directly to the ERBB2 amplicon but may represent genes co-regulated with *ERBB2*. For example, *NRG1* is known to regulate *ERBB2*, and chromosomal aberrations involving *NRG1* can mimic *ERBB2* amplification as in the case of the MDAMB175VII cell line ([*32*](#_ENREF_32)). These observations, closely aligned with prior expectations, suggest that the biological relevance of each feature and data type is driving their relative performance rather than technical differences between the data types/sets.

*Statistical methods* For inter-data correlation, the Spearman correlation coefficient was calculated at cell line level across genes passing the presence filter, and at gene level across the cell lines in common between the 2 data sets of focus.

Co-expression patterns between cell lines and tumor samples were investigated with use of the correlation-based coherence heatmap and the Jaccard similarity coefficient ([*33*](#_ENREF_33)). Coherence heatmaps were generated for the cell line panel and tumor data set separately. The Jaccard coefficient is defined as the number of gene pairs with the same correlation pattern in both coherence heatmaps, divided by the total number of gene pairs (only considering one triangular part of the heatmap). This coefficient ranges from 0 to 1, with values closer to 1 representing better similarity, values towards 0 representing anti-concordance, and a Jaccard coefficient of 0.5 corresponding to lack of any pattern between two random gene sets. The Fisher’s exact test was used to calculate how significantly the Jaccard coefficient differs from what is expected by chance (value of 0.5).

*Pathway overrepresentation analysis* For all compounds with test AUC > 0.7, the Cytoscape plug-in ClueGO ([*34*](#_ENREF_34)) was used for the assessment of overrepresented Gene Ontology (GO) Biological Process categories and pathways from KEGG and BioCarta in the molecular response signatures. Networks were constructed from the best performing signature per compound shown in **Table S5**, with RPPA proteins mapped to their unique gene symbol. Small signatures with <500 unique genes were first extended with genes not included in the signature but significantly associated with response with p-value after multiple testing correction with the Benjamini-Hochberg method (FDR) ([*35*](#_ENREF_35)) <0.2 (Wilcoxon rank sum test), obtained on each of the molecular data sets individually from which the signature was derived. The rationale for this was that only a subset of significant genes may have been included in the signature, whilst the full panel of significant genes should be considered for pathway analysis and biological interpretation. For the GO categories, redundancy was reduced by retaining the most representative category per parent-child relationship sharing gene sets with up to 1 gene difference. P-values were calculated with a two-sided minimal-likelihood test based on the hypergeometric distribution, with all genes included in the GO/KEGG/BioCarta database considered as reference set. The p-values were corrected for multiple testing with the false discovery rate (FDR) ([*35*](#_ENREF_35)). Significant pathways were selected based on two criteria: 1) FDR p-value < 0.05; and 2) presence of at least 5 genes from the gene list in the pathway. The analyses were based on the GO categories and KEGG pathways as of February 2, 2011 and the October 2008 version of the BioCarta database.

To exclude overrepresented GO categories and pathways due to subtype specific response, pathway analysis was additionally performed on the differentially expressed gene lists (FDR p-value < 0.1) for luminal vs. non-luminal, basal vs. non-basal, claudin-low vs. non-claudin-low, and ERBB2-amplified vs. non-ERBB2-amplified, obtained from the combined set of U133A, exon array (all features), RNAseq (all features), methylation, SNP6 and RPPA data. Out of 44,473 features, 2,920 unique genes were significant for luminal vs. non-luminal; 1,166 unique genes were significantly associated with basal; 1,958 unique genes with claudin-low; and 65 unique genes with ERBB2-amplification. Among those unique genes, 1,057 pathways were significantly associated with luminal with FDR p-value < 0.05, 657 with basal, 198 with claudin-low, and 9 with the ERBB2-amplified subtype.

Supplementary Results:

*Assessment of cell line signal in tumor samples* Before applying any of the cell line derived signatures to tumor samples, we verified for U133A, exon array and methylation whether the features obtained from the cell line data after filtering were detectable and did vary in clinical tumor samples. As can be seen in **Table S12**, features from U133A and the exon array that passed the variance and presence filter in the cell lines were present in the majority of breast cancer tumor samples, with 84-91% of features expressed above background in at least 20% of tumors. Those features also showed sufficient variation, with COV above 0.3 in 94.3-99.6% of features. This also held for the methylation CpG loci passing the variance filter, with COV exceeding 0.3 for 77.6% of the loci.

*Inter-data relationships* We first assessed correlations between the expression data sets, between expression and copy number, and between expression and methylation after reduction of the data sets to features passing the presence filter. For U133A vs. exon array, the Spearman correlation coefficient at cell line level across the 4,576 genes passing the presence filter in both data sets was on average 0.60 for 47 common cell lines. For U133A vs. RNAseq, the average correlation was 0.68 for 36 common cell lines across 4,831 genes. Correlation increased to 0.77 on average for 44 common cell lines when considering exon array vs. RNAseq with 8,879 genes in common. Association trends at gene level were similar, with correlation coefficients equal to 0.71, 0.58 and 0.68, respectively. Copy number ratios relative to normal sample arrays and gene expression were positively correlated: 0.20 at cell line level and 0.41 at gene level for U133A; 0.22 at cell line level and 0.44 at gene level for the exon array; 0.18 at cell line level and 0.35 at gene level for RNAseq. Promoter methylation and gene expression were on average negatively correlated as expected: -0.16 at cell line level and -0.12 at gene level for U133A; -0.19 at cell line level and -0.15 at gene level for the exon array; -0.25 at cell line level and -0.10 at gene level for RNAseq. Each distribution of correlation coefficient significantly deviated from zero (one-sample t-test, p-value < 0.0001).

We further explored the data sets as a function of copy number aberrations. First, copy number level of the 124 genes that passed variance filter for SNP6 were correlated with the corresponding gene levels - when measured - for each expression platform separately. After FDR correction with a significance level of 0.05, 9 out of 41 genes measured on the U133A platform (22%) showed a significant concordance between their genomic and transcriptomic profile. The same held for 25/69 (36%) genes for exon array and 26/66 (39%) genes for RNAseq. The percentage of genes with related copy number and expression (22-39%) is higher than was the case for mutation with expression (1/8 genes, 12%). Around half of these genes were amplified and half of the genes deleted in a subset of the cell lines. An overview of these genes with high correlation between SNP6 and expression is given in **Table S4**. The sets of amplified genes were highly overrepresented with genes from the ERBB2 amplicon. When restricted to the core amplicon of 10 genes, 3 of those appeared in the 9-gene list of U133A (33%), 8 in the 25-gene list of exon array (32%) and 9 in the 26-gene list of RNAseq (35%). When considering 24 genes in the core amplicon and its 500kb flanking regions (both up- and down-stream), no additional genes were found with U133A, while the number of high correlation genes in this region increased to 10 (40%) and 12 (46%) for exon array and RNAseq, respectively.

*Robust predictors of drug response are found at all levels of the genome.* With this many data types available on a single set of samples, we were well positioned to assess whether particular technologies or molecular data types consistently out-perform others in the prediction of drug sensitivity. To obtain a ranking of the importance of the molecular data sets, we built classifiers on individual data sets, and compared prediction performance to one another.

The RPPA dataset contains measurements from only 70 well-known (phospho)proteins. To account for this difference in number of measurements between the genome-wide datasets and the RPPA dataset, we reduced the genome-wide data sets to 55 unique genes that correspond to the 70 (phospho)proteins. For RNAseq, exon array and methylation, we included data on all 55 genes and the corresponding features from the other levels (transcripts, exons, etc.) that passed stringent filtering based on the full set of cell lines. This resulted in 271 features for RNAseq, 529 for exon array, and 108 for methylation. **Table S6a** and **S6c** show the ranking of the data sets according to the independent classifiers obtained with LS-SVM and Random Forests, respectively. For the LS-SVM classifiers in **Table S6a**, methylation provided the highest AUC for about a quarter of the compounds (22), followed by RNAseq, SNP6, exon array and RPPA, performing best for 16 to 14 compounds. For 8 compounds, prediction was most accurate with inclusion of U133A. Results were confirmed with the Random Forests approach (**Table S6c**). The full combination of RPPA and reduced genome-wide datasets only yielded a higher AUC value than the best performing individual data source with Random Forests for gefinitib.

While the strategy adopted above represents a ‘real world’ situation where we would use as many variables as possible, in actuality different data types have very different numbers of potential predictors, which may influence their relative performances. Therefore, we built classifiers on a combined set of the most significant features from each of the considered data sets, with inclusion of an equal number of features per data set for an unbiased comparison. The first combined set consisted of the 100 most differentially expressed features per genome-wide data set, with redundancy reduction in RNAseq, exon array and methylation to the feature per gene with the most significant p-value. Differential expression was obtained from the 29 common cell lines, with the statistical method depending on variable type (Wilcoxon rank sum test for continuous variables, Kruskal Wallis test for ordinal variables, Fisher’s exact test for nominal variables). The second combined set, with inclusion of RPPA, was performed on the set of 55 genes corresponding to the 70 RPPA proteins, with redundancy reduction in RPPA, RNAseq, exon array and methylation to the feature per gene with the best p-value based on the 27 common cell lines.

The appearance of features from the different data sets in the top 100 of the overall feature ranking obtained with LS-SVM is shown in **Table S6e**. When restricted to the RPPA-based gene set, a similar number of features from all data sets was observed among the 100 highest ranked features, varying from 11% for SNP6 to 25% for exon array. This points towards similar relevance of the data sets when focused on a particular set of genes. When looking at the genome-wide data sets, on average 43% of the features in the top 100 were from the RNAseq data set, and around a quarter of features from both exon array and methylation (29% and 24%, respectively). Only an average of 3.2% and 0.8% of the highest ranked features were from U133A and SNP6, respectively.

*Validation against other cell line datasets* A recent publication presented a large multi-tumor-type cell line panel assayed against various cancer therapies and raised the possibility of an independent validation set for our own breast-cancer-specific cell line based predictors. The Cancer Cell Line Encyclopedia (CCLE) ([*36*](#_ENREF_36)) has assembled a set of 1001 cell lines and has assayed 504 of these against at least one of 24 compounds. The cell lines were profiled for DNA copy number (Affymetrix SNP6 array), mRNA expression level (Affymetrix U133Plus2 array) and mutation status of >1,600 genes (targeted massively parallel sequencing). The entire set includes 59 breast lines of which 29 have data for at least one drug as well as copy number and expression status allowing prediction of drug sensitivity by our software. Overall, our breast cancer panel includes 37 lines not represented in CCLE and is missing only 12 of their 59. The majority of CCLE lines with drug, expression and CNV data were included in our training dataset (24/29) and thus represent technical replicates more than biological replicates. Of 24 drugs assayed in CCLE, only 9 overlap with the 90 drugs assayed in our study and of those, we only produced high-confidence predictors for 4 drugs (erlotinib, sorafenib, lapatinib, paclitaxel). Extracting data for these 4 drugs from CCLE, we found that only lapatinib and paclitaxel had substantial variation in drug sensitivity (measured as IC50). Based on these data, we could not confidently separate resistant from sensitive cell lines, and so determined these data to be unsuitable for validation purposes.

As part of another study primarily specifically focused on HER2+ lines (manuscript in preparation), additional drug response measurements subsequently became available for 11 lines, for at least one of six drugs, where those cell line and drug combinations were not used in training. This resulted in a total of 41 new cell line/drug combinations with which to assess accuracy: 11 cell lines for BIBW2992, 8 cell lines for Lapatinib, 8 cell lines for Rapamycin, 8 cell lines for GSK2126458A, 5 cell lines for Iressa and 1 cell line for GSK2141795c. We compared predicted probability of sensitivity determined by the signatures for these drugs to actual sensitivity determined by comparing measured GI50 to the same mean GI50 cutoffs used to classify training data (**Table S14**). Overall accuracy was good with 78.0% of predicted sensitivity class in agreement with measured sensitivity class. However there was considerable variability in performance with 100% (8/8 correct) for Lapatinib, 100% (1/1) for GSK2141795c, 90.9% (10/11) for BIBW2992, 87.5% (7/8) for Rapamycin, 60% (3/5) for Iressa, and 37.5% (3/8) for GSK2126458A. Overall, these results are promising and indicate that sensitivity of additional lines, profiled in the same manner, can be accurately predicted. However, we must also point out that while these results are for an independent set of cell line drug responses, the dataset is both small and biased towards the HER2+ subtype and HER2 inhibitors. Thus, while promising, these results are possibly limited in generalizability to other subtypes or compound types. Additional validation experiments for the signatures developed in this study await the creation of additional cell line datasets or molecular profiling of patient tumors with appropriate treatment response data.

*Patient response prediction toolbox in R* In the context of personalized medicine, it would be of interest to estimate a breast cancer patient’s likelihood of response to a broad set of available therapeutic compounds. We provide software (at Synapse: <https://www.synapse.org/#!Synapse:syn2179898> and at GitHub: https://github.com/obigriffith/Rtoolbox) through which a list of 90 compounds is ranked for a patient according to predicted response based on gene expression, copy number and/or methylation data, upon availability for that patient. Best results are expected if data are provided from platforms used for the cell line data, on which the classifiers were built (Affymetrix GeneChip Human Genome U133A, Affymetrix Genome-Wide Human SNP Array 6.0 and Illumina’s Infinium HumanMethylation BeadChip for expression, copy number and methylation respectively). In this case, the input files per patient should be one or more of the following: U133A CEL-file, SNP6 CEL-file, and a tab-delimited file with the proportion of methylated DNA at each measured CpG locus. The U133A CEL-file is normalized with the U133A CEL-files of the 48 core cell lines, whilst the SNP6 CEL-file is segmented using the same breakpoints as obtained in the cell line panel. However, we also provide a platform agnostic toolbox in R that is independent of the platforms used to obtain expression, copy number and/or methylation data. In this case, the input files per patient should be one or more of the following: a tab-delimited file with gene symbol and expression level, a tab-delimited file with gene symbol and copy number level, and a tab-delimited file with the proportion of methylated DNA at each measured CpG locus. Both the expression and copy number data are first quantile normalized to the cell line distributions. Predicted response for each patient is provided for those compounds with AUC>0.6 for the best cell line-derived predictor based on any or all of the input data and with provided patient data on a sufficient number of predictor/model variables. The latter requires a weighted percent of model variables (WPMV) of at least 80% calculated as the sum of input (user-provided) variable ranks (where most important variables receive the largest rank value) divided by the sum of all model variable ranks.

The platform agnostic toolbox was applied to the subset of 306 TCGA samples with Agilent expression, Affymetrix SNP6 copy number values and Illumina methylation data. For each compound, the best performing model was utilized (LS-SVM or RF with any combination of expression, copy number and methylation data). Compounds without a model AUC > 0.7 and compounds without at least a single patient with a predicted probability of response > 0.65 were excluded, leaving 22 compounds of interest. The resulting probabilities of response are summarized for all 22 compounds and 306 patients along with their clinical features and subtype in the heatmap shown in **Fig. 3**. Association between predicted sensitivity and clinical variables (ER, PR, ERBB2, T, N, M, subtype, and AJCC Stage) was determined by Wilcox or ANOVA where appropriate. P-values were corrected for multiple testing by the Benjamini-Hochberg method using the ‘multtest’ package in R. The results of all statistical tests are summarized in **Table S9** and a few key associations illustrated in **Fig. S9**. All compounds but two (gefitinib and NU6102) were significantly associated with subtype (p-values = 1.5e-70 to 0.02) (**Table S9**). For example, luminal and ERBB2-amplified cell lines were typically sensitive to AKT inhibition while basal and claudin-low cell lines were resistant. In the TCGA data, consistently more luminal and *ERBB2*-amplified tumor samples were predicted to respond to this compound compared to basal samples (p-value 3.9e-6, **Table S5**). All compounds were also significantly associated with at least one of ERBB2, ER or PR status. Lapatinib, BIBW2992 and tamoxifen showed the expected association between sensitivity and ERBB2 or ER status, respectively (**Fig. S9a-c**). Given the expected association between tamoxifen response and ER status it was observed that the default response probability cutoff of 0.5 was likely inappropriate for some compounds. Therefore, compound-specific cutoffs were chosen objectively from the distributions of probabilities using the ‘mclust’ package in R. Mclust attempts to separate the probabilities based on a Gaussian mixture model (mclust was allowed to determine the best model). If only a single cluster was detected cutoffs were set at the first and third quartile. If two clusters were detected one cutoff was set between the clusters and the median value of the larger distribution used for the second cutoff. For three distributions cutoffs were selected as the midpoints between each cluster. For cases of 4 or more clusters, the clusters were manually grouped. The final result was the division of patients for the 22 compounds into three classes: sensitive, intermediate, and resistant. **Table S10** summarizes all cutoffs and the numbers of patients in each class that resulted. An example of the probability distributions and cutoffs chosen after mixed model clustering are shown for 5-FU in **Fig. S8**. To allow better comparison between compounds with different thresholds for sensitivity, values were rescaled by simple linear conversion from the range of all (unscaled) values within a response class to 0 to 0.333 for resistant values, 0.333 to 0.666 for intermediate values, and 0.666 to 1 for sensitive values.

Supplementary Tables:

**Table S1.** Overview of 84 cell lines with subtype information and available data. GI_50_ values for 90 therapeutic compounds are provided for 70/84 cell lines included in all analyses.

**Table S2.** Processed Reverse Protein Lysate Array (RPPA) intensity data for 70 (phospho)proteins with fully validated antibodies in 49 cell lines. See **Supplementary Methods** for data processing details.

**Table S3.** GI_50_ dichotomization threshold for each compound, defined as the mean GI_50_ for the 48 core cell lines.

**Table S4**

1. Overview of genes with good correlation with FDR p-value < 0.05 between SNP6 and U133A expression. 22% of genes in copy number aberration regions show a significant concordance between their genomic and transcriptomic profile after multiple testing correction.

| **Gene** | **Spearman corr coeff** | **P-value** | **FDR p-value** | **ERBB2 core amplicon** | **ERBB2 core amplicon with 500kb flanking regions (up- and down-stream)** | **Deletion / amplification** |
| --- | --- | --- | --- | --- | --- | --- |
| BCAS1 | 0.463 | 0.0015 | 0.0079 |  |  | Amplification |
| CDKN2A | 0.818 | 0 | 0 |  |  | Deletion |
| ERBB2 | 0.680 | 3.8e-07 | 3.1e-06 | Y | Y | Amplification |
| GRB7 | 0.601 | 1.6e-05 | 9.5e-05 | Y | Y | Amplification |
| GSTT1 | 0.748 | 6.2e-08 | 6.9e-07 |  |  | Deletion |
| MTAP | 0.725 | 2.6e-08 | 5.2e-07 |  |  | Deletion |
| SMAD4 | 0.710 | 6.7e-08 | 6.9e-07 |  |  | Deletion |
| STARD3 | 0.610 | 1.1e-05 | 7.6e-05 | Y | Y | Amplification |
| WNK1 | 0.432 | 0.0034 | 0.0154 |  |  | Deletion |

1. Overview of genes with good correlation with FDR p-value < 0.05 between SNP6 and exon array expression. 36% of genes in copy number aberration regions show a significant concordance between their genomic and transcriptomic profile after multiple testing correction.

| **Gene** | **Spearman corr coeff** | **P-value** | **FDR p-value** | **ERBB2 core amplicon** | **ERBB2 core amplicon with 500kb flanking regions (up- and down-stream)** | **Deletion / amplification** |
| --- | --- | --- | --- | --- | --- | --- |
| ADAM32 | 0.393 | 0.0046 | 0.0158 |  |  | Amplification |
| ANKRD15 | 0.531 | 7.8e-05 | 0.0004 |  |  | Deletion |
| BCAS1 | 0.488 | 0.0003 | 0.0012 |  |  | Amplification |
| C17orf37 | 0.779 | 1.7e-11 | 3.0e-10 | Y | Y | Amplification |
| C9orf53 | 0.331 | 0.0177 | 0.0490 |  |  | Deletion |
| CDKN2A | 0.819 | 0 | 0 |  |  | Deletion |
| CDKN2B | 0.699 | 5.1e-08 | 5.8e-07 |  |  | Deletion |
| CRKRS | 0.719 | 2.7e-09 | 3.8e-08 |  | Y | Amplification |
| ELAVL2 | 0.351 | 0.0116 | 0.0349 |  |  | Deletion |
| ERBB2 | 0.649 | 2.7e-07 | 2.6e-06 | Y | Y | Amplification |
| FBXL20 | 0.627 | 8.3e-07 | 5.7e-06 |  | Y | Amplification |
| GBP3 | 0.585 | 6.6e-06 | 3.8e-05 |  |  | Deletion |
| GRB7 | 0.611 | 1.9e-06 | 1.2e-05 | Y | Y | Amplification |
| GSTT1 | 0.632 | 6.4e-07 | 4.9e-06 |  |  | Deletion |
| MTAP | 0.883 | 9.8e-18 | 3.4e-16 |  |  | Deletion |
| PERLD1 | 0.536 | 5.0e-05 | 0.0002 | Y | Y | Amplification |
| PNMT | 0.456 | 0.0008 | 0.0029 | Y | Y | Amplification |
| PPP1R1B | 0.435 | 0.0014 | 0.0052 | Y | Y | Amplification |
| RHD | 0.342 | 0.0145 | 0.0418 |  |  | Deletion |
| SLC25A24 | 0.549 | 3.0e-05 | 0.0002 |  |  | Deletion |
| SMAD4 | 0.833 | 3.3e-14 | 7.5e-13 |  |  | Deletion + amplification |
| STARD3 | 0.634 | 6.0e-07 | 4.9e-06 | Y | Y | Amplification |
| TCAP | 0.367 | 0.0080 | 0.0263 | Y | Y | Amplification |
| ZNF28 | 0.492 | 0.0002 | 0.0011 |  |  | Deletion |
| ZNF462 | 0.362 | 0.0090 | 0.0281 |  |  | Deletion |

1. Overview of genes with good correlation with FDR p-value < 0.05 between SNP6 and RNAseq expression. 39% of genes in copy number aberration regions show a significant concordance between their genomic and transcriptomic profile after multiple testing correction.

| **Gene** | **Spearman corr coeff** | **P-value** | **FDR p-value** | **ERBB2 core amplicon** | **ERBB2 core amplicon with 500kb flanking regions (up- and down-stream)** | **Deletion / amplification** |
| --- | --- | --- | --- | --- | --- | --- |
| ADAM32 | 0.512 | 0.0002 | 0.0006 |  |  | Amplification |
| BCAS1 | 0.676 | 1.4e-07 | 5.6e-07 |  |  | Amplification |
| C17orf37 | 0.852 | 1.6e-14 | 5.3e-13 | Y | Y | Amplification |
| CDKN2A | 0.746 | 1.2e-09 | 9.8e-09 |  |  | Deletion |
| CDKN2B | 0.647 | 6.6e-07 | 2.6e-06 |  |  | Deletion |
| CRKRS | 0.732 | 3.5e-09 | 2.3e-08 |  | Y | Amplification |
| DMRTA1 | 0.390 | 0.0061 | 0.0161 |  |  | Deletion |
| ELAVL2 | 0.412 | 0.0036 | 0.0100 |  |  | Deletion |
| ERBB2 | 0.806 | 5.0e-12 | 8.3e-11 | Y | Y | Amplification |
| FBXL20 | 0.710 | 1.6e-08 | 7.8e-08 |  | Y | Amplification |
| GBP3 | 0.482 | 0.0005 | 0.0016 |  |  | Deletion |
| GRB7 | 0.875 | 4.3e-16 | 2.8e-14 | Y | Y | Amplification |
| GSTM1 | 0.360 | 0.0119 | 0.0301 |  |  | Amplification |
| GSTT1 | 0.710 | 1.6e-08 | 7.8e-08 |  |  | Deletion |
| HLA-DRB5 | 0.548 | 5.4e-05 | 0.0002 |  |  | Deletion + amplification |
| HSF2BP | 0.544 | 6.5e-05 | 0.0002 |  |  | Deletion |
| MTAP | 0.746 | 1.2e-09 | 9.8e-09 |  |  | Deletion |
| NEUROD2 | 0.681 | 1.0e-07 | 4.4e-07 | Y | Y | Amplification |
| PERLD1 | 0.740 | 1.8e-09 | 1.3e-08 | Y | Y | Amplification |
| PNMT | 0.723 | 6.6e-09 | 4.0e-08 | Y | Y | Amplification |
| PPARBP | 0.818 | 1.3e-12 | 2.8e-11 |  | Y | Amplification |
| PPP1R1B | 0.632 | 1.4e-06 | 5.3e-06 | Y | Y | Amplification |
| RHD | 0.447 | 0.0016 | 0.0046 |  |  | Deletion |
| SMAD4 | 0.710 | 1.6e-08 | 7.8e-08 |  |  | Deletion + amplification |
| STARD3 | 0.762 | 3.2e-10 | 3.5e-09 | Y | Y | Amplification |
| TCAP | 0.798 | 1.1e-11 | 1.5e-10 | Y | Y | Amplification |

**Table S5.** Overview of the best LS-SVM/RF model for all 90 therapeutic compounds with comparison to the LS-SVM AUC based on subtype and ERBB2 status. For the subset of 51 therapeutic compounds with test AUC exceeding 0.7, additional information is provided on clinical trial status, comparison of GI_50_ with TGI, validation results of the cell line signal in the TCGA tumor samples, and most significant non-subtype related KEGG/BioCarta pathways from **Table S7**.

**Table S6a.** Data type ranking of the importance of the molecular datasets by comparison of prediction performance of LS-SVM classifiers built on individual data sets and their combination, with and without inclusion of RPPA data.

|  | **RPPA-subset comparison** | | | **Genome-wide comparison** | | |
| --- | --- | --- | --- | --- | --- | --- |
| **Data type** | **Avg. AUC rank (std)** | **Median AUC rank (25th-75th percentile)** | **# compounds for which data type yields highest AUC** | **Avg. AUC rank (std)** | **Median AUC rank (25th-75th percentile)** | **# compounds for which data type yields highest AUC** |
| RNAseq | 3.69 (1.92) | 4 [2-5] | 16 | 2.82 (1.53) | 3 [2-4] | 22 |
| Exon array | 3.79 (1.90) | 4 [2-5] | 15 | 3.13 (1.64) | 3 [2-4] | 20 |
| Methylation | 3.78 (2.31) | 4 [2-6] | 22 | 3.94 (1.74) | 4 [2-5] | 12 |
| RPPA | 3.83 (2.08) | 3.5 [2-6] | 14 | n/a | n/a | n/a |
| U133A | 4.24 (1.79) | 4 [3-6] | 8 | 3.32 (1.72) | 3 [2-5] | 17 |
| SNP6 | 4.60 (2.35) | 5.5 [2-7] | 15 | 4.42 (1.98) | 5 [3-6] | 18 |
| Full combination | 4.07 (1.42) | 4 [3-5] | 0 | 3.36 (1.01) | 3 [3 4] | 1 |

Note: For each compound, the individual data sets and full combination were assigned a rank in decreasing order of AUC from 1 to 6 or 7 (for the genome-wide and RPPA-subset comparison, respectively). The average and median AUC rank for each data set across the 90 compounds are shown.

**Table S6b.** Data type comparison and importance for independent LS-SVM classifiers: examples of compounds for which (most) datasets give similar results or for which one dataset performs better with AUC increase of at least 0.1 (shown in bold).

| AUC | **GSK 461364** | **CGC-11047** | **Lapatinib** | **VX-680** | **Carboplatin** | **BIBW2992** | **Evero-limus** |
| --- | --- | --- | --- | --- | --- | --- | --- |
| U133A | **0.849** | **0.735** | **0.868** | 0.718 | 0.442 | 0.641 | 0.547 |
| Exon array | **0.884** | **0.710** | **0.860** | **0.813** | 0.475 | **0.760** | 0.702 |
| RNAseq | **0.823** | **0.814** | **0.888** | 0.678 | **0.887** | **0.770** | 0.687 |
| SNP6 | 0.706 | 0.575 | **0.914** | 0.390 | 0.460 | **0.858** | 0.386 |
| Methylation | 0.700 | **0.754** | 0.580 | 0.621 | 0.547 | 0.488 | **0.831** |
| RPPA | **0.854** | n/a | n/a | n/a | 0.481 | **0.768** | 0.634 |
| Full combination | **0.846** | **0.804** | **0.874** | 0.731 | 0.834 | 0.737 | 0.731 |

**Table S6c.** Data type ranking of the importance of the molecular datasets by comparison of prediction performance of Random Forests classifiers built on individual data sets and their combination, with and without inclusion of RPPA data.

|  | **RPPA-subset comparison** | | | **Genome-wide comparison** | | |
| --- | --- | --- | --- | --- | --- | --- |
| **Data type** | **Avg. AUC rank (std)** | **Median AUC rank (25th-75th percentile)** | **# compounds for which data type yields highest AUC** | **Avg. AUC rank (std)** | **Median AUC rank (25th-75th percentile)** | **# compounds for which data type yields highest AUC** |
| RNAseq | 3.57 (1.93) | 3 [2-5] | 16 | 2.90 (1.54) | 3 [1-4] | 25 |
| Exon array | 3.62 (1.76) | 4 [2-5] | 14 | 3.14 (1.63) | 3 [2-5] | 22 |
| Methylation | 3.88 (2.29) | 4 [1-6] | 23 | 3.87 (1.92) | 4 [2-6] | 18 |
| RPPA | 3.89 (2.20) | 4 [2-6] | 15 | n/a | n/a | n/a |
| U133A | 4.42 (1.90) | 5 [3-6] | 8 | 3.74 (1.60) | 4 [2-5] | 8 |
| SNP6 | 4.67 (2.27) | 5 [2-7] | 13 | 4.07 (2.01) | 5 [2-6] | 15 |
| Full combination | 3.96 (1.30) | 4 [3-5] | 1 | 3.28 (1.14) | 3 [2-4] | 2 |

Note: For each compound, the individual data sets and full combination were assigned a rank in decreasing order of AUC from 1 to 6 or 7 (for the genome-wide and RPPA-subset comparison, respectively). The average and median AUC rank for each data set across the 90 compounds are shown.

**Table S6d.** Data type comparison and importance for independent RF classifiers: examples of compounds for which (most) datasets give similar results or for which one dataset performs better with AUC increase of at least 0.1 (shown in bold).

| AUC | **Lapatinib** | **GSK2126458** | **CGC-11047** | **TGX-221** | **Docetaxel** | **Bortezomib** |
| --- | --- | --- | --- | --- | --- | --- |
| U133A | 0.847 | 0.808 | 0.760 | **0.844** | **0.828** | 0.719 |
| Exon array | **0.900** | **0.914** | 0.770 | 0.740 | **0.752** | 0.731 |
| RNAseq | **0.967** | **0.849** | **0.883** | **0.747** | 0.675 | 0.706 |
| SNP6 | **0.880** | 0.788 | 0.674 | 0.669 | 0.521 | 0.719 |
| Methylation | 0.653 | 0.773 | **0.827** | **0.792** | **0.763** | 0.594 |
| RPPA | **0.947** | n/a | n/a | **0.799** | n/a | **0.869** |
| Full combination | **0.900** | **0.864** | **0.847** | 0.734 | 0.710 | 0.731 |

**Table S6e.** Data type comparison and importance based on the average appearance of data types in the top 100 of ranked features.

| **Data type** | **Avg. appearance of data types in the top 100 of ranked features (%), restricted to genes corresponding to the 70 protein RPPA set** | **Avg. appearance of data types in the top 100 of ranked features (%), restricted to the top 100 features per genome-wide data set (RPPA excluded)** |
| --- | --- | --- |
| Exon array | 24.9 | 29.5 |
| RNAseq | 19.5 | 42.7 |
| Methylation | 17.3 | 23.8 |
| U133A | 12.2 | 3.2 |
| SNP6 | 10.6 | 0.8 |
| RPPA | 15.5 | **n/a** |

**Table S7.** List of significant non-subtype specific GO categories and KEGG/BioCarta pathways with FDR p-value < 0.05. Per category/pathway, information is provided on FDR p-value and the number of signature genes, percentage of signature genes and list of signature genes that are part of this category/pathway. Significant pathways associated with both drug response and transcriptional subtype were excluded, to capture biology underlying each compound’s mechanism of action.**Table S8.** Performance for “splice-specific” response predictors (RF) with an AUC increase > 0.05 when comparing all transcript features to gene-level values alone.

| **Data set** | **Compound** | **# cell lines** | **AUC (All feat.)** | **AUC (gene level)** | **Δ AUC (all-gene)** |
| --- | --- | --- | --- | --- | --- |
| RNAseq | BEZ235 | 36 | 0.607 | 0.449 | 0.158 |
|  | 17-AAG | 36 | 0.604 | 0.498 | 0.105 |
|  | GSK1838705A (IGF1R) | 37 | 0.652 | 0.548 | 0.104 |
|  | BIBW2992 | 31 | 0.791 | 0.705 | 0.085 |
|  | GSK923295 | 35 | 0.652 | 0.576 | 0.076 |
|  | CGC-11047 | 37 | 0.807 | 0.737 | 0.070 |
|  | CPT-11(FD) | 36 | 0.616 | 0.548 | 0.068 |
|  | Geldanamycin | 37 | 0.608 | 0.553 | 0.056 |
|  | FTase inhibitor I | 36 | 0.742 | 0.687 | 0.055 |
|  | Lapatinib | 35 | 0.973 | 0.918 | 0.054 |
|  | SAHA (Vorinostat) | 37 | 0.818 | 0.765 | 0.053 |
| Exon-array | BIBW2992 | 35 | 0.714 | 0.543 | 0.170 |
|  | Lapatinib | 40 | 0.875 | 0.809 | 0.066 |

**Table S9.** Statistical association between clinical variables and predicted response for 306 TCGA patients with expression, methylation and copy number data available. For each compound, the best performing model was utilized (LSSVM or RF with any combination of expression, copy number and methylation data).

| **Compound** | **ER** | **PR** | **ERBB2** | **T** | **N** | **M** | **TP53** | **PIK3CA** | **Stage** | **Subtype** |
| --- | --- | --- | --- | --- | --- | --- | --- | --- | --- | --- |
| **5-FU** | 1.06E-14 | 9.31E-05 | 9.12E-03 | 1.09E-02 | 2.53E-02 | 5.82E-02 | 7.49E-04 | 8.61E-01 | 8.69E-02 | 1.70E-28 |
| **AG1478** | 3.38E-01 | 8.67E-01 | 1.23E-06 | 3.53E-01 | 3.38E-01 | 5.83E-01 | 3.23E-01 | 5.89E-01 | 8.35E-01 | 4.48E-04 |
| **AKT1-2 inhibitor** | 2.29E-11 | 5.06E-04 | 1.90E-09 | 2.20E-01 | 1.09E-02 | 3.09E-01 | 9.12E-03 | 4.27E-01 | 4.59E-01 | 1.98E-66 |
| **BIBW2992** | 8.08E-01 | 4.33E-01 | 3.05E-17 | 2.06E-02 | 1.47E-01 | 5.18E-01 | 2.69E-02 | 9.43E-01 | 2.01E-01 | 2.13E-23 |
| **Bortezomib** | 7.82E-29 | 7.09E-17 | 3.07E-01 | 8.44E-01 | 9.48E-02 | 3.07E-01 | 2.17E-14 | 6.38E-01 | 8.08E-02 | 5.31E-70 |
| **Cisplatin** | 1.74E-23 | 8.14E-15 | 4.48E-01 | 9.79E-01 | 3.12E-01 | 2.69E-01 | 2.85E-14 | 7.74E-01 | 2.43E-01 | 5.03E-56 |
| **Etoposide** | 9.23E-01 | 9.15E-01 | 7.41E-03 | 4.36E-01 | 7.57E-01 | 9.36E-02 | 1.89E-01 | 2.12E-01 | 9.67E-02 | 7.09E-04 |
| **Fascaplysin** | 7.40E-01 | 9.02E-01 | 8.91E-04 | 7.91E-01 | 1.31E-02 | 3.81E-01 | 1.00E-02 | 3.36E-02 | 3.09E-01 | 2.17E-02 |
| **Gefitinib** | 6.54E-01 | 9.45E-01 | 2.92E-05 | 7.78E-01 | 7.58E-02 | 9.74E-01 | 2.01E-01 | 2.30E-01 | 7.75E-01 | 2.38E-01 |
| **GSK461364A** | 7.70E-24 | 1.44E-14 | 8.44E-01 | 4.21E-01 | 1.09E-02 | 1.31E-01 | 2.08E-12 | 5.13E-01 | 2.69E-01 | 1.85E-70 |
| **GSK2119563A** | 5.32E-08 | 1.33E-03 | 7.49E-04 | 8.17E-01 | 3.00E-03 | 5.23E-01 | 3.31E-02 | 9.27E-03 | 5.68E-01 | 1.29E-31 |
| **GSK2126458A** | 6.78E-11 | 4.95E-04 | 5.95E-07 | 2.12E-01 | 7.73E-03 | 1.60E-01 | 4.41E-03 | 1.47E-01 | 3.07E-01 | 6.05E-62 |
| **GSK2141795** | 7.09E-17 | 8.78E-09 | 2.43E-01 | 2.19E-01 | 3.93E-01 | 2.61E-01 | 5.80E-07 | 1.44E-01 | 8.35E-01 | 3.61E-25 |
| **GSK1059615B** | 2.35E-02 | 3.17E-01 | 1.63E-05 | 3.45E-02 | 4.36E-02 | 2.20E-01 | 5.74E-01 | 4.59E-01 | 1.62E-01 | 1.34E-11 |
| **GSK1120212B** | 1.72E-01 | 2.43E-01 | 5.63E-03 | 1.44E-01 | 6.53E-01 | 3.07E-01 | 4.17E-01 | 3.47E-03 | 4.59E-01 | 5.25E-12 |
| **Ixabepilone** | 4.47E-14 | 1.25E-06 | 2.68E-01 | 1.34E-02 | 2.05E-01 | 3.83E-02 | 4.75E-05 | 5.09E-01 | 2.12E-01 | 2.21E-23 |
| **Lapatinib** | 3.19E-02 | 8.79E-03 | 1.34E-14 | 3.88E-01 | 4.36E-02 | 2.18E-01 | 3.07E-01 | 6.02E-01 | 2.27E-01 | 2.13E-23 |
| **NU6102** | 1.21E-01 | 4.69E-02 | 6.53E-01 | 1.78E-01 | 9.45E-01 | 6.04E-01 | 1.75E-02 | 8.44E-01 | 5.24E-01 | 4.33E-01 |
| **Nutlin 3a** | 6.80E-07 | 7.09E-04 | 8.87E-01 | 8.85E-01 | 5.14E-01 | 5.83E-01 | 3.70E-04 | 1.13E-02 | 2.20E-01 | 1.03E-04 |
| **Paclitaxel** | 5.38E-05 | 1.61E-02 | 3.93E-01 | 7.47E-01 | 9.23E-01 | 6.48E-01 | 8.61E-07 | 3.43E-02 | 4.59E-01 | 1.69E-06 |
| **PF-3084014** | 5.02E-15 | 2.31E-10 | 2.33E-02 | 8.50E-01 | 5.83E-01 | 8.18E-01 | 1.23E-15 | 3.92E-01 | 2.05E-02 | 7.08E-20 |
| **Tamoxifen** | 1.38E-15 | 2.61E-10 | 3.93E-01 | 8.44E-01 | 5.18E-01 | 4.17E-01 | 3.54E-07 | 5.72E-02 | 8.85E-01 | 4.43E-20 |

NOTE: Values are BH corrected p-values from Wilcox test (ER, PR, ERBB2, T, N, M, TP53, PIK3CA) or ANOVA (Stage, Subtype). Significant p-values are shown in red.

**Table S10.** Resistant/intermediate/sensitive cut-offs for 22 compounds with model AUC > 0.7 and at least one patient with probability of response > 0.65. Cutoff value 1 separates patients considered resistant from intermediate. Cutoff value 2 separates patients considered intermediate from sensitive. The % value for each group indicates the percentage of total patients (N=306) in each group.

| **Compound** | **Cutoff 1** | **Cutoff 2** | **Low %** | **Int %** | **High %** |
| --- | --- | --- | --- | --- | --- |
| 5-FU | 0.503 | 0.746 | 38.2 | 37.9 | 23.9 |
| AG1478 (EGFR) | 0.102 | 0.251 | 37.6 | 31.7 | 30.7 |
| Sigma AKT1-2i | 0.492 | 0.582 | 19.3 | 40.2 | 40.5 |
| BIBW2992 (ERBB2) | 0.105 | 0.285 | 57.2 | 27.1 | 15.7 |
| Bortezomib | 0.562 | 0.599 | 38.9 | 37.6 | 23.5 |
| Cisplatin | 0.564 | 0.599 | 37.6 | 36.9 | 25.5 |
| Etoposide | 0.173 | 0.300 | 36.3 | 35.9 | 27.8 |
| Fascaplysin | 0.114 | 0.355 | 45.1 | 27.5 | 27.5 |
| Gefitinib | 0.085 | 0.232 | 44.1 | 29.1 | 26.8 |
| GSK1120212B (MEK) | 0.261 | 0.879 | 20.9 | 49.3 | 29.7 |
| GSK461364A (PLK) | 0.465 | 0.545 | 39.2 | 38.9 | 21.9 |
| GSK2119563A (PI3K alpha) | 0.547 | 0.598 | 13.1 | 43.1 | 43.8 |
| GSK2126458A (PI3K, pan) | 0.580 | 0.648 | 17.0 | 40.8 | 42.2 |
| GSK2141795 (AKT) | 0.548 | 0.609 | 25.2 | 49.7 | 25.2 |
| GSK1059615B (PI3K) | 0.758 | 0.935 | 49.3 | 26.1 | 24.5 |
| Lapatinib | 0.177 | 0.614 | 64.7 | 19.6 | 15.7 |
| Ixabepilone | 0.578 | 0.598 | 41.5 | 29.1 | 29.4 |
| NU6102 (CDK/CCNB) | 0.527 | 0.643 | 25.2 | 49.7 | 25.2 |
| Nutlin 3a | 0.152 | 0.830 | 12.4 | 52.0 | 35.6 |
| PF-3084014 | 0.573 | 0.622 | 25.5 | 49.3 | 25.2 |
| Paclitaxel | 0.547 | 0.576 | 25.2 | 49.3 | 25.5 |
| Tamoxifen | 0.064 | 0.275 | 10.1 | 55.2 | 34.6 |

**Table S11.** Compound response signatures for the 22 compounds featured in **Fig. 5** with model AUC > 0.7 and at least one patient from the TCGA set of 306 tumor samples with expression, copy number and methylation data available with probability of response > 0.65.

**Table S12.** Presence and variance of filtered features from U133A and exon array cell line data in tumor samples ([*13*](#_ENREF_13)*,* [*14*](#_ENREF_14)) (TCGA data obtained from <http://tcga-data.nci.nih.gov/tcga/)>. Features from U133A and the exon array that passed the variance and presence filter in the cell lines were present in the majority of breast cancer tumor samples.

| **Platform** | **Gene set** | **Criterion** | **COV (for the variance criterion) and percentage of tumor samples with gene expression above background (for the presence criterion) across the gene set** | | |
| --- | --- | --- | --- | --- | --- |
|  |  |  | **Mean** | **Median** | **25th-75th percentile** |
| U133A | 840 probes | presence* | 66.9 | 80.7 | 38.7-97.3 |
| U133A | 840 probes | variance (COV) | 0.734 | 0.614 | 0.485-0.837 |
| Exon | 1,338 genes | presence* | 61.7 | 67.9 | 32.1-94.1 |
| Exon | 1,338 genes | variance (COV) | 0.695 | 0.582 | 0.440-0.828 |
| Exon | 1,572 transcripts | presence* | 64.8 | 72.6 | 36.9-96.4 |
| Exon | 1,572 transcripts | variance (COV) | 0.734 | 0.619 | 0.465-0.863 |
| Exon | 10,289 exons | presence* | 70.2 | 79.8 | 47.6-97.6 |
| Exon | 10,289 exons | variance (COV) | 0.994 | 0.872 | 0.641-1.201 |
| Methylation | 7,263 CpG loci | variance (COV) | 0.605 | 0.528 | 0.316-0.838 |

* background level of 195 in U133A tumor data and 70 in exon array tumor data (adjusted background level due to shift in distribution)

**Table S13.** Summary of 167 predictors in Random Forests classifier for Lapatinib (all data types, optimal predictor number).

| **Data set** | **Gene** | **Feature level(s)** | **Best RF variable importance Rank** |
| --- | --- | --- | --- |
| **SNP6** | STARD3 | Gene | 1 |
|  | TCAP | Gene | 2 |
|  | ZNFN1A3 | Gene | 3 |
|  | PERLD1 | Gene | 4 |
|  | PNMT | Gene | 6 |
|  | NEUROD2 | Gene | 9 |
|  | PPP1R1B | Gene | 10 |
|  | C17orf37 | Gene | 12 |
|  | ERBB2 | Gene | 13 |
|  | GRB7 | Gene | 14 |
|  | CRKRS | Gene | 133 |
| **RNAseq** | GRB7 | Exon (15), Boundary (1), Junction (17), Transcript (2), Gene | 5 |
|  | CADPS2* | Exon (1), Boundary (2) | 15 |
|  | COL5A1* | Exon (6), Junction (6) | 21 |
|  | AL391099.12* | Exon (1), Transcript (1), Gene | 22 |
|  | GUCY1A3* | Exon (1) | 26 |
|  | TUBB4* | Exon (1), Transcript (1) | 28 |
|  | NRG1* | Exon (2), Boundary (1) | 30 |
|  | CRISPLD1* | Exon (1) | 34 |
|  | STARD3 | Junction (2) | 36 |
|  | RBM24* | Exon (1) | 37 |
|  | MYO15B* | Boundary (1) | 39 |
|  | NA* | Intergenic (2) | 41 |
|  | GAS6* | Junction (1) | 72 |
|  | PPP1R1B | Exon (2), Junction (2) | 79 |
|  | ERBB2 | Exon (2), Junction (5) | 107 |
|  | C17orf37 | Exon (2), Junction (1), Transcript (1) | 109 |
|  | ACY3* | Exon (1) | 117 |
|  | DLC1* | Exon (1) | 132 |
|  | PERLD1 | Exon (2), Junction (3), Boundary (1) | 147 |
| **Exon array** | ERBB2 | Exon (35), Transcript (1), Gene | 7 |
|  | GRB7 | Gene | 11 |
|  | PERLD1 | Exon (6), Transcript (1), Gene | 16 |
|  | PRSS8* | Gene | 40 |
|  | STARD3 | Exon (9) | 88 |
|  | C17orf37 | Exon (3), Transcript (1) | 130 |
|  | ST6GAL1* | Exon (1) | 144 |
| **U133A** | GRB7 | Gene | 19 |
|  | ERBB2 | Gene | 111 |
| **Meth** | FLJ27365* | Promoter | 102 |
| **RPPA** | ERBB2p1248 | Protein | 115 |

* Genes not directly located within the ERBB2 amplicon region.

**Table S14.** Validation dataset and results. The excel file includes in one worksheet an overview of 11 cell lines with subtype information and GI_50_ values for 7 therapeutic compounds. In the second worksheet are results of running these cell lines through predictors for the drugs for which they have measured drug response values. Predicted sensitivity is compared to measured sensitivity to assess accuracy of signatures.

**Supplementary Figures:**


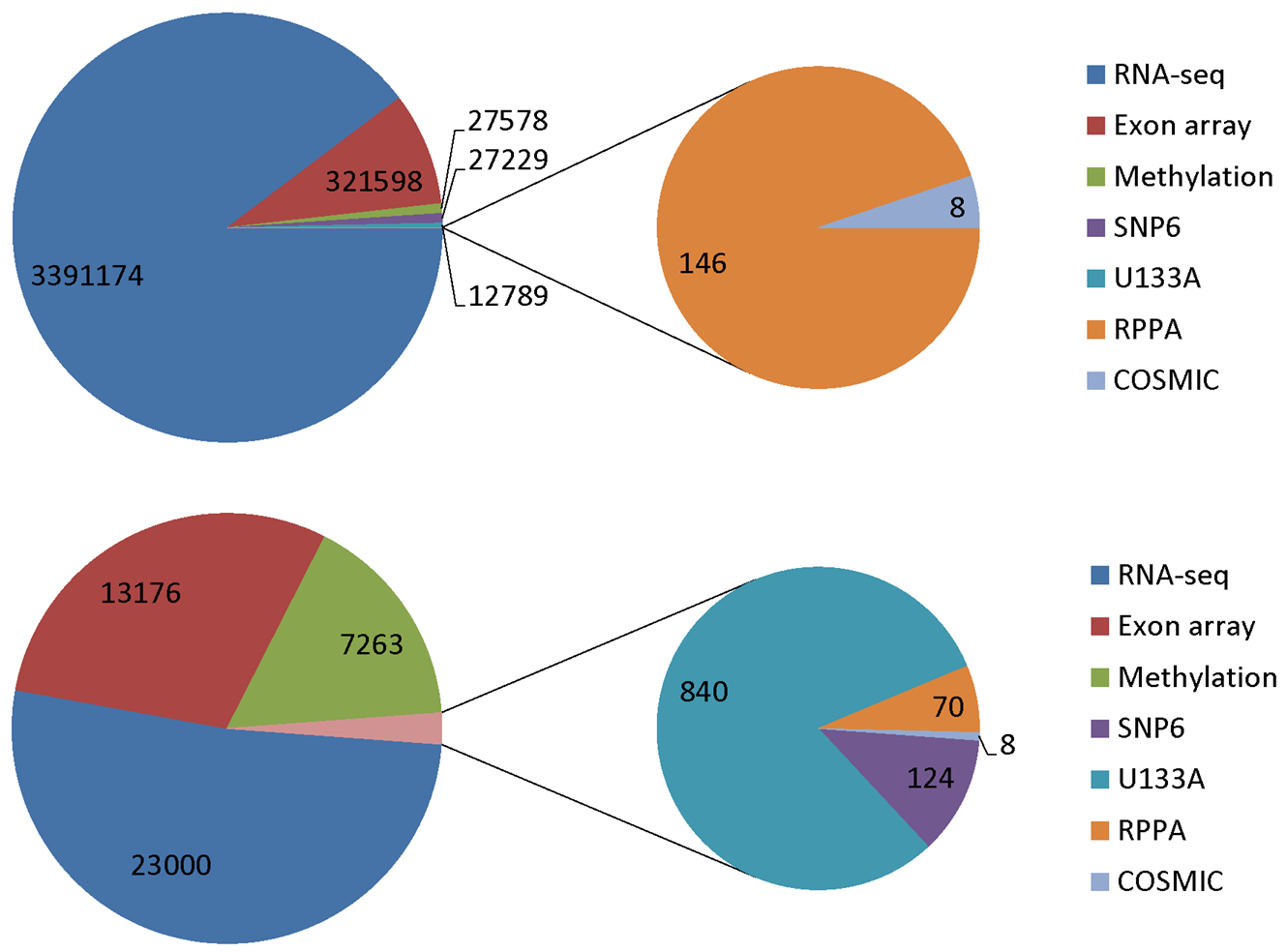


**Fig. S1.** Data summary in terms of number of features before (top) and after (bottom) data-type-specific reduction and unsupervised filtering based on variance and signal detection above background.

**
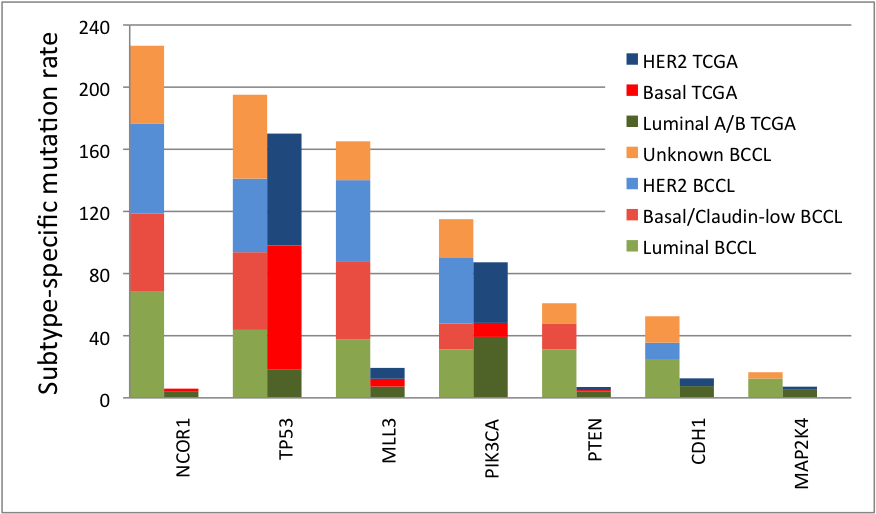
**

**Fig. S2.** Overview of the mutation prevalence in the cell line panel and TCGA data set for the list of 7 common coding variants detected by TCGA, with a distinction between luminal (green), basal (red) and ERBB2-enriched (blue). Cell lines with unknown subtype are displayed in orange. To make the subtypes comparable, luminal A and B were grouped into luminal for the TCGA data set, whilst basal and claudin-low cell lines were grouped into basal. The mutation rate in TCGA and the cell line panel shows a similar distribution across the subtypes.

**Fig. S3.** Comparison of the best LS-SVM and RF models for the 90 compounds, sorted according to highest AUC obtained with either model.


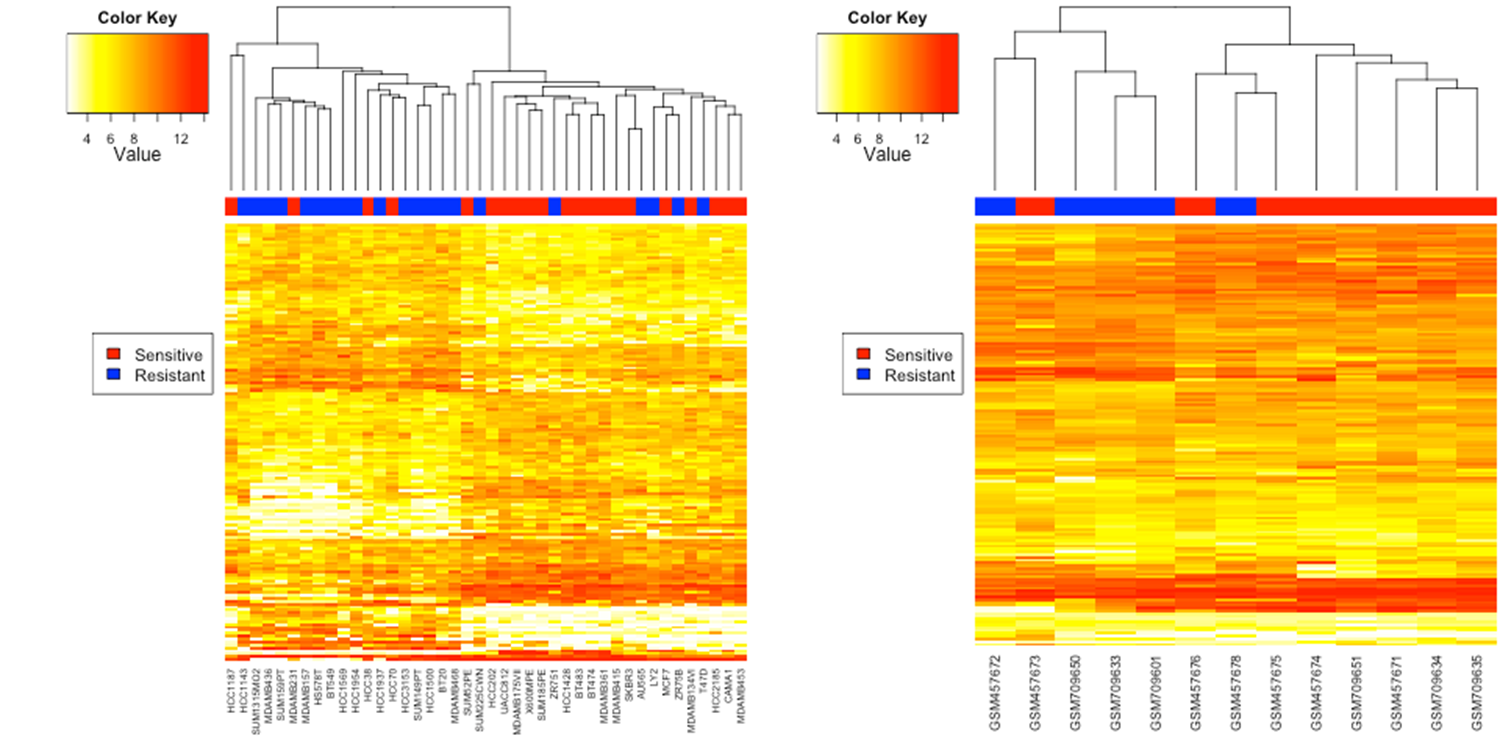


**Fig. S4.** Validation of the cell line signature for vorinostat in tumor samples grown in 3D: heatmap of the 150-gene signature for vorinostat in the cell line panel (left) and 13 tumor samples treated with valproic acid (right). 7/8 sensitive samples (87.5%) and 4/5 resistant samples (80%) are classified correctly with a probability threshold of 0.5 for response dichotomization.


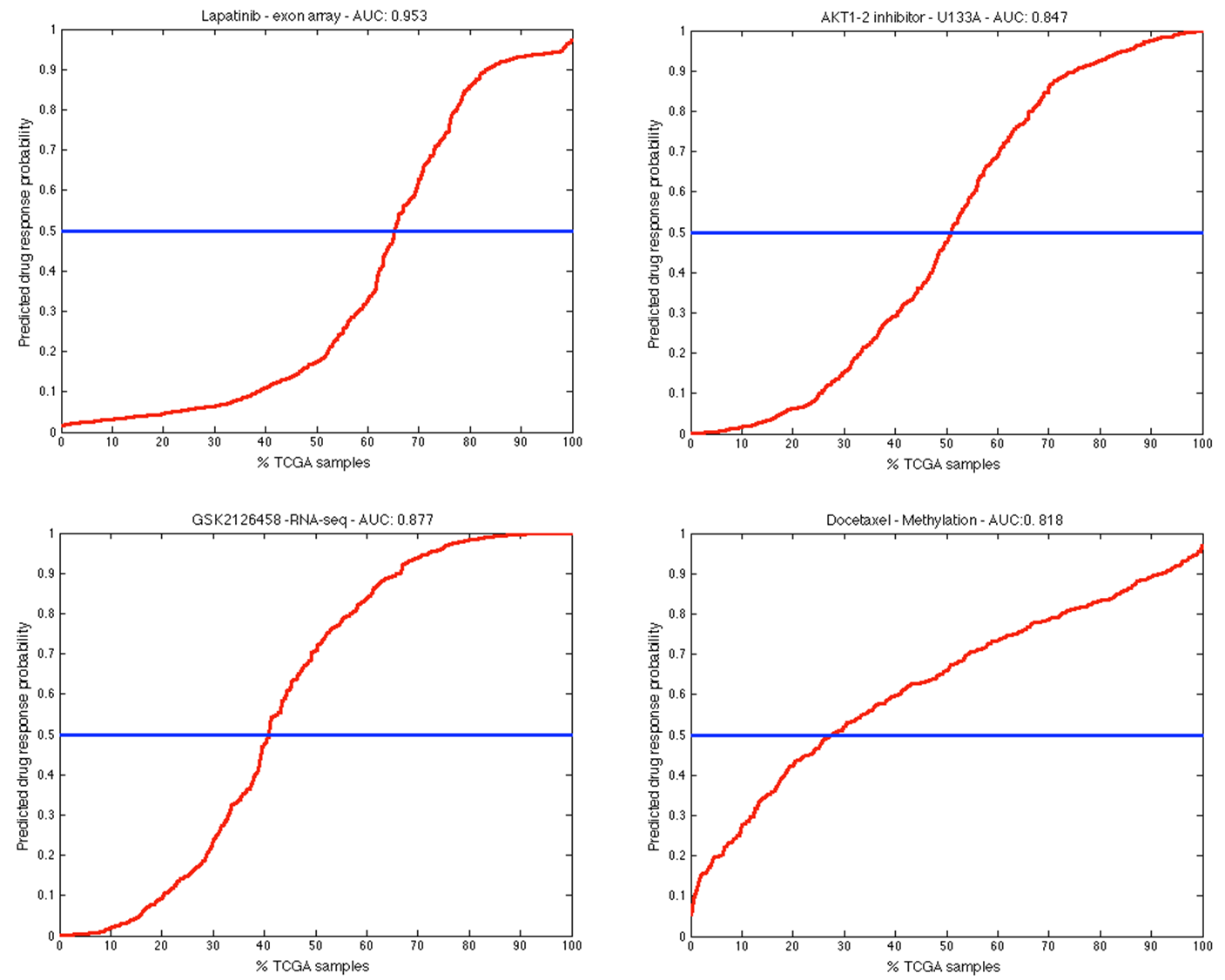


**Fig. S5.** Predicted probability of response of TCGA tumor samples to compounds lapatinib, sigma AKT1-2 inhibitor, GSK2126458 and docetaxel. The TCGA tumor samples are ordered according to increasing probability of response.


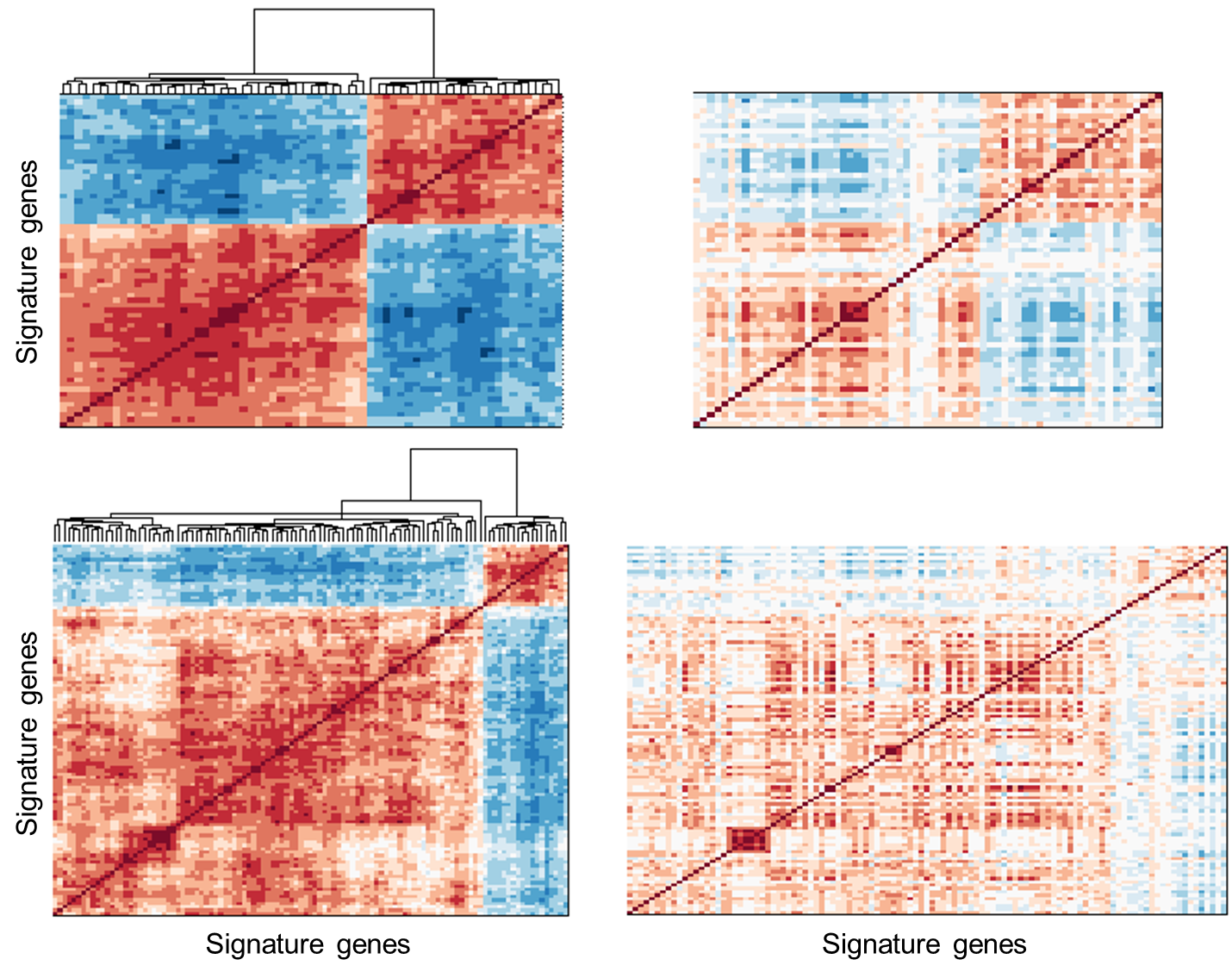


**Fig. S6.** Correlation-based coherence heatmap for 2 cell line-derived gene signatures. **Top** Coherence among 67 genes of the U133A signature for the sigma AKT1-2 inhibitor in the cell lines (left) and TCGA tumor samples (right) (Jaccard coefficient = 0.85; p-value < 0.0001); **Bottom** Coherence among 109 genes of the RNAseq signature for everolimus in the cell lines (left) and TCGA tumor samples (right) (Jaccard coefficient = 0.79; p-value < 0.0001).

**Fig. S7.** Comparison of the best model per dataset for the 90 compounds, sorted according to highest AUC obtained with either model (LS-SVM or random forest). For RNAseq and exon array, the highest AUC is shown among models built on gene-level data only vs. all features (exons, junctions, etc).


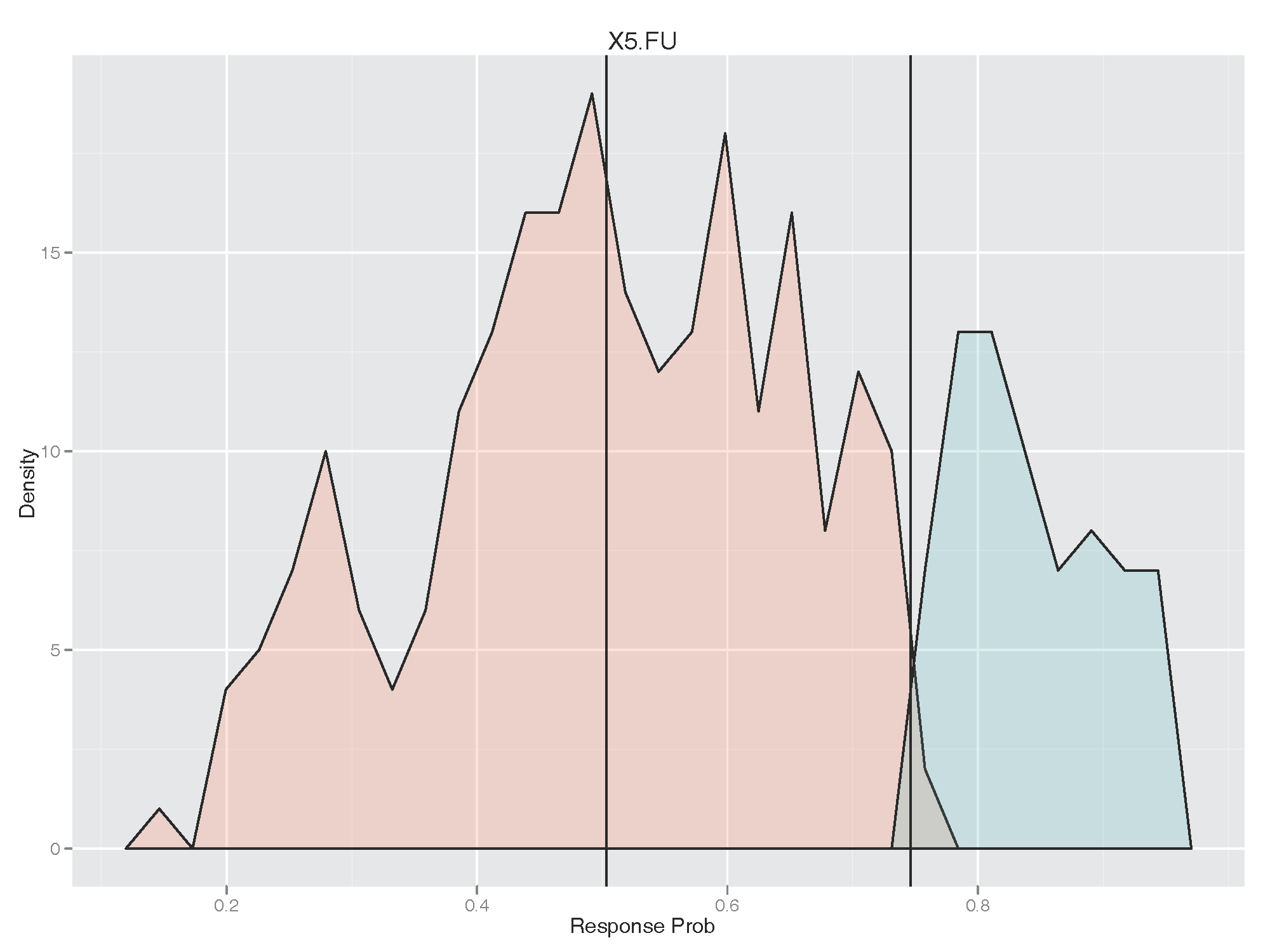


**Fig. S8.** Distributions of response probabilities for 5-FU determined by mixed model clustering and used for cut-off selection. With a cut-off of 0.74, 23.9% of TCGA tumor samples were predicted to respond to 5-FU (**Table S10**).


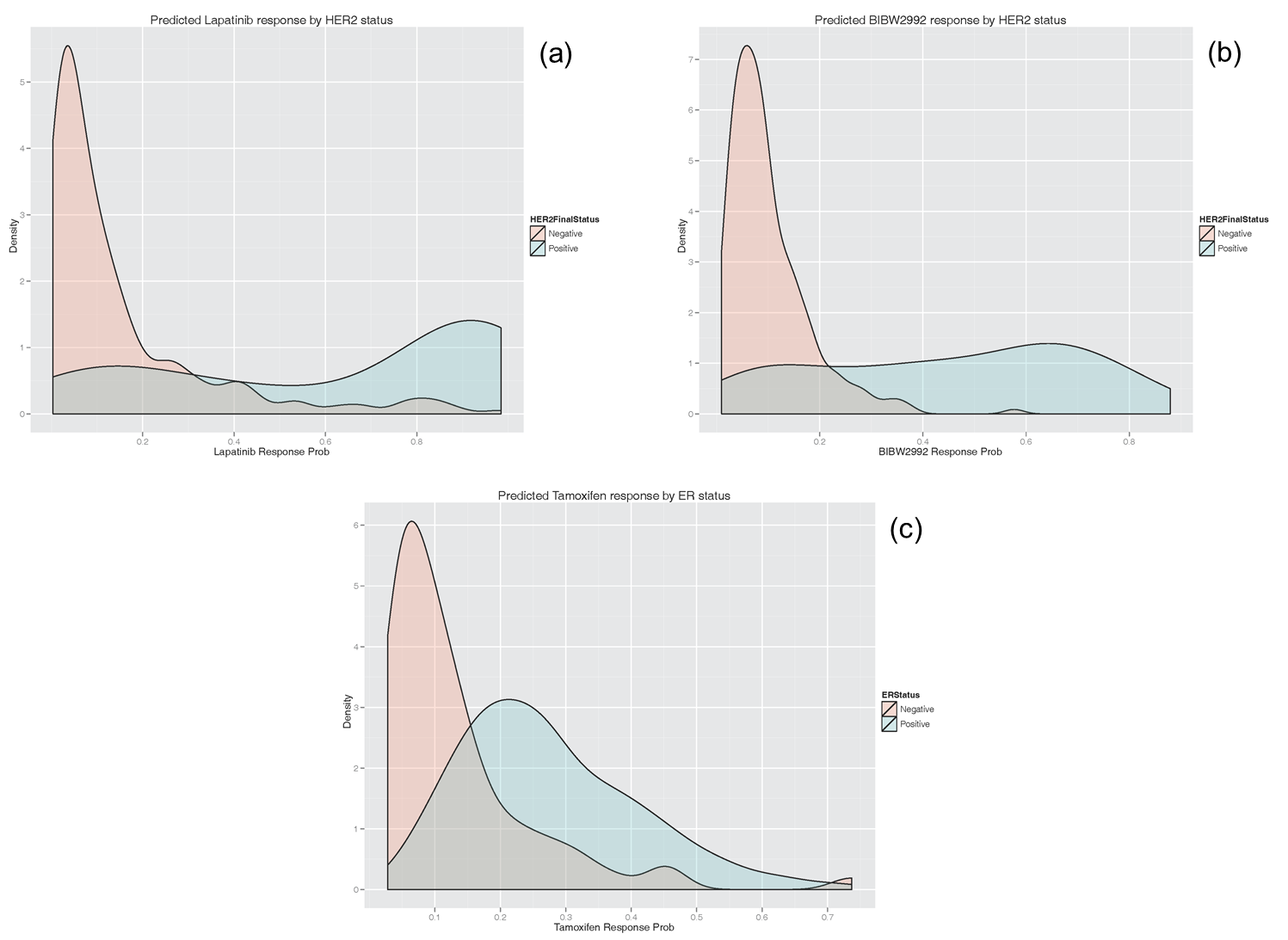


**Fig. S9.** Association between (a) response to Lapatinib and ERBB2 status, (b) response to BIBW2992 and ERBB2 status, and (c) response to Tamoxifen and ER status for 306 TCGA patients with expression, methylation and copy number data available.


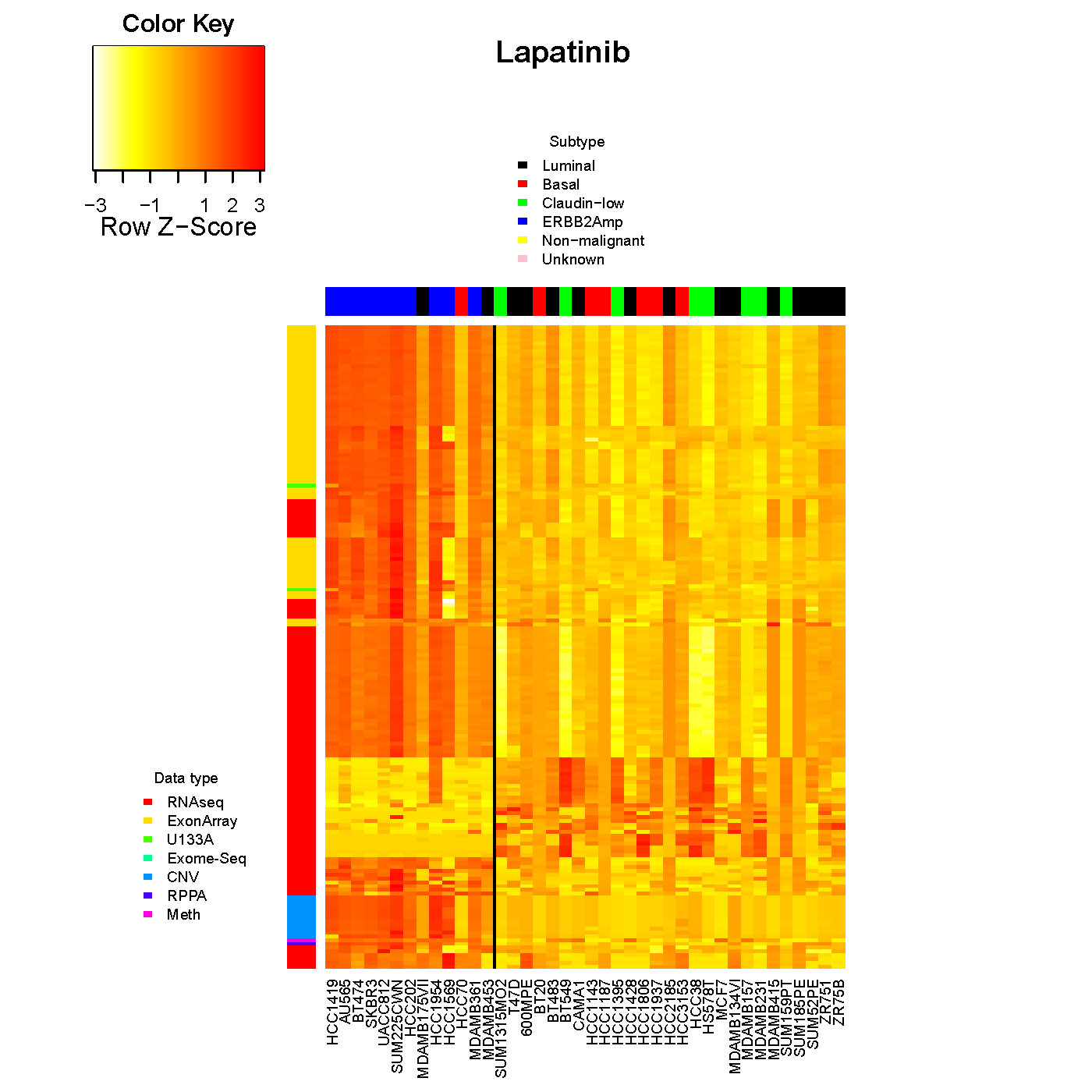


**Fig. S10.** Heatmap of the 167 highest ranked features for Lapatinib, obtained with random forest applied to the full set of molecular data.

**References**

1. L. M. Heiser *et al.*, Subtype and pathway specific responses to anticancer compounds in breast cancer. *Proc Natl Acad Sci U S A*, (Oct 14, 2011).

2. H. Bengtsson, R. Irizarry, B. Carvalho, T. P. Speed, Estimation and assessment of raw copy numbers at the single locus level. *Bioinformatics* **24**, 759 (Mar 15, 2008).

3. H. Bengtsson, P. Wirapati, T. P. Speed, A single-array preprocessing method for estimating full-resolution raw copy numbers from all Affymetrix genotyping arrays including GenomeWideSNP 5 & 6. *Bioinformatics* **25**, 2149 (Sep 1, 2009).

4. E. S. Venkatraman, A. B. Olshen, A faster circular binary segmentation algorithm for the analysis of array CGH data. *Bioinformatics* **23**, 657 (Mar 15, 2007).

5. O. Troyanskaya *et al.*, Missing value estimation methods for DNA microarrays. *Bioinformatics* **17**, 520 (Jun, 2001).

6. M. Dai *et al.*, Evolving gene/transcript definitions significantly alter the interpretation of GeneChip data. *Nucleic acids research* **33**, e175 (2005).

7. The Cancer Genome Atlas Network, Integrated genomic analyses of ovarian carcinoma. *Nature* **474**, 609 (Jun 30, 2011).

8. M. Griffith *et al.*, Alternative expression analysis by RNA sequencing. *Nat Methods* **7**, 843 (Oct, 2010).

9. M. J. Fackler *et al.*, Genome-Wide Methylation Analysis Identifies Genes Specific to Breast Cancer Hormone Receptor Status and Risk of Recurrence. *Cancer Res*, (Aug 8, 2011).

10. R. Tibes *et al.*, Reverse phase protein array: validation of a novel proteomic technology and utility for analysis of primary leukemia specimens and hematopoietic stem cells. *Mol Cancer Ther* **5**, 2512 (Oct, 2006).

11. H. Li, J. Ruan, R. Durbin, Mapping short DNA sequencing reads and calling variants using mapping quality scores. *Genome Res* **18**, 1851 (Nov, 2008).

12. S. Chiaretti *et al.*, Gene expression profile of adult T-cell acute lymphocytic leukemia identifies distinct subsets of patients with different response to therapy and survival. *Blood* **103**, 2771 (Apr 1, 2004).

13. M. Lukk *et al.*, A global map of human gene expression. *Nat Biotechnol* **28**, 322 (Apr, 2010).

14. E. Lin *et al.*, Exon array profiling detects EML4-ALK fusion in breast, colorectal, and non-small cell lung cancers. *Mol Cancer Res* **7**, 1466 (Sep, 2009).

15. J. S. Parker *et al.*, Supervised risk predictor of breast cancer based on intrinsic subtypes. *J Clin Oncol* **27**, 1160 (Mar 10, 2009).

16. M. J. Bissell, Architecture Is the Message: The role of extracellular matrix and 3-D structure in tissue-specific gene expression and breast cancer. *The Pezcoller Foundation journal : news from the Pezcoller Foundation world* **16**, 2 (Oct, 2007).

17. P. A. Kenny *et al.*, The morphologies of breast cancer cell lines in three-dimensional assays correlate with their profiles of gene expression. *Molecular oncology* **1**, 84 (Jun, 2007).

18. M. J. Bissell, W. C. Hines, Why don't we get more cancer? A proposed role of the microenvironment in restraining cancer progression. *Nat Med* **17**, 320 (Mar, 2011).

19. A. L. Cohen *et al.*, A pharmacogenomic method for individualized prediction of drug sensitivity. *Mol Syst Biol* **7**, 513 (Jul 19, 2011).

20. S. Loi *et al.*, Definition of clinically distinct molecular subtypes in estrogen receptor-positive breast carcinomas through genomic grade. *J Clin Oncol* **25**, 1239 (Apr 1, 2007).

21. Y. Zhang *et al.*, The 76-gene signature defines high-risk patients that benefit from adjuvant tamoxifen therapy. *Breast Cancer Res Treat* **116**, 303 (Jul, 2009).

22. W. F. Symmans *et al.*, Genomic index of sensitivity to endocrine therapy for breast cancer. *J Clin Oncol* **28**, 4111 (Sep 20, 2010).

23. C. Sotiriou *et al.*, Gene expression profiling in breast cancer: understanding the molecular basis of histologic grade to improve prognosis. *J Natl Cancer Inst* **98**, 262 (Feb 15, 2006).

24. T. Barrett *et al.*, NCBI GEO: archive for functional genomics data sets--10 years on. *Nucleic Acids Res* **39**, D1005 (Jan, 2011).

25. J. Suykens, J. Vandewalle, Least Squares Support Vector Machine classifiers. *Neural Process Lett* **9**, 293 (1999).

26. J. Suykens, T. Van Gestel, J. De Brabanter, B. De Moor, J. Vandewalle, *Least Squares Support Vector Machines*. (World Scientific, Singapore, 2002).

27. B. Hanczar *et al.*, Small-sample precision of ROC-related estimates. *Bioinformatics* **26**, 822 (Mar 15, 2010).

28. G. Cawley, paper presented at the International Joint Conference on Neural Networks, 2006.

29. A. Daemen *et al.*, Improved modeling of clinical data with kernel methods. *Artificial intelligence in medicine* **54**, 103 (Feb, 2012).

30. H. Lin, C. Lin, R. Weng, A note on Platt's probabilistic outputs for support vector machines. *Mach Learn* **68**, 267 (2007).

31. A. Daemen *et al.*, A kernel-based integration of genome-wide data for clinical decision support. *Genome medicine* **1**, 39 (2009).

32. X. Liu, E. Baker, H. J. Eyre, G. R. Sutherland, M. Zhou, Gamma-heregulin: a fusion gene of DOC-4 and neuregulin-1 derived from a chromosome translocation. *Oncogene* **18**, 7110 (Nov 25, 1999).

33. C. Van Rijsbergen, *Information retrieval*. (Butterworth, London, 1979).

34. G. Bindea *et al.*, ClueGO: a Cytoscape plug-in to decipher functionally grouped gene ontology and pathway annotation networks. *Bioinformatics* **25**, 1091 (Apr 15, 2009).

35. Y. Benjamini, Y. Hochberg, Controlling the false discovery rate: a practical and powerful approach to multiple testing. *J Royal Stat Soc B* **57**, 289 (1995).

36. J. Barretina *et al.*, The Cancer Cell Line Encyclopedia enables predictive modelling of anticancer drug sensitivity. *Nature* **483**, 603 (Mar 29, 2012).
